# Integrative transcriptomics identifies an 18-gene candidate blood-accessible biomarker panel for longitudinal monitoring of spaceflight-induced muscle atrophy

**DOI:** 10.1371/journal.pone.0358130

**Published:** 2026-10-07

**Authors:** Jae Ho Shim, Ji Yeon Lee, Jihye Han, Hyeon Soo Kim

**Affiliations:** Department of Anatomy, Korea University College of Medicine, Seoul, Republic of Korea; Gyeongsang National University, KOREA, REPUBLIC OF

## Abstract

Skeletal muscle atrophy is a critical physiological risk during long-duration spaceflight, yet candidate blood-accessible transcriptomic biomarkers for longitudinal monitoring remain unavailable. We developed an integrative multi-mission framework combining a primary discovery transcriptome from THP-1 monocytic cells exposed to International Space Station (ISS) microgravity (GEO: Sci. Adv. 2026 (https://doi.org/10.1126/sciadv.adw7832, Data S1-S10); NASA OSDR: Al-Ahmadi et al. 2026; n = 8 spaceflight, 8 ground control) — yielding 4,522 spaceflight-differentially expressed genes (SDEGs) — with a PRISMA-compliant meta-analysis of 46 verified spaceflight studies, producing a 726-gene consensus set. Cross-referencing these datasets identified 103 intersection genes; application of our Integrated Concordance Score (ICS) nominated an 18-gene candidate biomarker panel nominated across six external missions. Orthogonal cross-tissue concordance analysis using six C2C12 myotube simulated-microgravity datasets demonstrated Pearson r = 0.79 (95% CI 0.52–0.92, p < 0.001), with a conserved stress module achieving r = 0.88; complementary concordance in human primary skeletal myotubes confirmed tri-model directional concordance for 13 stress-module genes. Gold-tier biomarkers (TNNT3, TRIM63, ACTC1, IGF2; ICS ≥ 8.0) showed ≥83% multi-mission concordance. Temporal modelling informs a preliminary two-tier fortnightly/monthly monitoring framework aligned with measured saturation kinetics, and the prioritised panel converges on coordinated ubiquitin-proteasome activation, AKT–mTOR–FOXO3a remodelling, E2F suppression, and sarcomeric reprogramming. Integrated drug–gene analysis identified bimagrumab combined with leucine as the top predicted prioritised countermeasure pair (computational Bliss = 2.8). These findings establish a proposed candidate blood-accessible biomarker framework for periodic muscle health surveillance during long-duration human spaceflight, available prospective validation in astronaut blood samples. The unit of analysis was the analytical contrast (spaceflight vs. matched ground control), not the publication. Cross-platform harmonization was performed via gene-level mapping and rank-based normalization.

## Introduction

Prolonged microgravity induces progressive skeletal muscle atrophy, with reductions of 10–20% in muscle mass documented across ISS missions [1–3]. This loss accompanies slow-to-fast fibre-type remodelling, reduced oxidative capacity, and impaired post-flight recovery [4–7]. Resistive exercise countermeasures partially mitigate but do not fully prevent atrophy in all crew members [8]. A critical operational gap is the absence of candidate blood-accessible molecular biomarkers for periodic longitudinal muscle health surveillance during flight. Existing approaches rely on ground-based imaging and dynamometry — operationally constrained and infrequent within ISS schedules [9,10]. Transcriptomic biomarkers from peripheral blood samples offer a practical alternative compatible with established RNA stabilisation protocols and ISS sample-return logistics [11].

Monocytic cells occupy the skeletal muscle microenvironment as muscle-resident macrophages and participate in atrophy-associated paracrine signalling, sharing conserved stress-response architecture — ubiquitin-proteasome system (UPS), mammalian target of rapamycin (mTOR), Forkhead box O (FOXO), and nuclear factor kappa-light-chain-enhancer of activated B cells (NF-κB) — with myofibres [12–14]. THP-1 human monocytic cells (ATCC TIB-202) are therefore well-suited as an ISS discovery platform, given their suspension-culture compatibility, cryopreservation tolerance (RNA integrity number [RIN] ≥ 8.5 post-ISS return), and full stress-pathway gene expression (S1 File, S1 Text). We refer to ISS-induced THP-1 differentially expressed genes as spaceflight-differentially expressed genes (SDEGs) throughout the manuscript [15]. Here we introduce the Integrated Concordance Score (ICS), a composite metric combining discovery-level evidence with cross-study consistency, and validate an 18-gene panel through cross-tissue concordance analysis in C2C12 myotubes [16], orthogonal confirmation in human primary skeletal myotubes, multi-mission concordance across six independent missions, and temporal modelling to inform a preliminary monitoring framework.

## Materials and methods

### Cell culture and ISS experiment

THP-1 cells (ATCC TIB-202) were maintained in RPMI-1640 supplemented with 10% heat-inactivated foetal bovine serum, 2 mM L-glutamine, and 0.05 mM 2-mercaptoethanol at 37 °C in 5% CO_2_. ISS culture comprised 14 days of microgravity exposure in NASA Biological Research in Canisters (BRIC) hardware aboard the ISS; RNA was stabilised in RNAlater immediately post-culture, and simultaneous ground-control cultures were maintained at NASA Johnson Space Center (JSC). No human-subject or animal experiments were performed; the study was exempt under JSC-IRB-2019–0847 (commercially available cell line).

### RNA sequencing and differential expression analysis

TruSeq Stranded mRNA libraries (Illumina) were sequenced on an Illumina NovaSeq 6000 (150 bp paired-end; ≥ 30 M reads per sample). Reads were aligned with STAR v2.7.10a to GRCh38.p13/GENCODE v38; gene-level quantification used featureCounts v2.0.1; differential expression analysis used DESeq2 v1.34.0 with apeglm log2 fold change (Log2FC) shrinkage, applying thresholds of false discovery rate (FDR) < 0.05 and |Log2FC| > 0.5 [17]. The sample size (n = 8 biological replicates per group) exceeds the minimum recommended for DESeq2-based differential expression at these thresholds. Raw data were deposited at GEO (Sci. Adv. 2026 (https://doi.org/10.1126/sciadv.adw7832, Data S1-S10)) and NASA OSDR (Al-Ahmadi et al. 2026).

### ICS meta-analysis

A PRISMA 2020-compliant systematic review was conducted (S1 File, Checklist 1) and identified 46 PubMed-verified empirical transcriptomics studies (S13 Table (S1 Table), verified registry) published between 2000 and 2024. Random-effects models with REML estimation were fitted using metafor v3.8.1. The Integrated Concordance Score was defined as ICS = 0.6 × S4522 + 0.4 × S726, where S4522 = 0.6 × Log2FC_norm + 0.4 × (−log10FDR_norm) and S726 = 0.5 × N_studies,norm + 0.25 × DisGeNET_norm + 0.25 × Category_norm. All sub-scores were min–max normalised to [0,10] across 103 intersection genes. Tier thresholds were Gold (ICS ≥ 8.0), High (7.0–7.9), and Moderate (6.0–6.9). Sensitivity analysis used four alternative weighting configurations (A–D); bootstrap validation used n = 10,000 BCa iterations; permutation testing used n = 1,000 shuffled gene–disease annotation datasets.

### C2C12 and human primary myotube cross-tissue concordance

Cross-platform and cross-cohort validation refined the spaceflight-associated muscle signature from the initial THP-1 discovery set to independent murine, astronaut, and orthogonal muscle-cell validation systems (Fig 3). Differential-expression and recurrence filtering retained a core candidate set carried forward into the ICS framework, with directional concordance preserved for representative upregulated contractile and calcium-handling genes (TNNT3, TNNI3, MYH6, MYH7, CASQ2, RYR1) and downregulated cytoskeletal, growth-signalling, and cell-cycle markers (TPM2, IGF2, PCNA, MCM2) across independent in vivo and astronaut-derived datasets. Temporal trajectories indicated progressive change during spaceflight or unloading rather than transient single-time-point shifts, supporting longitudinal monitoring applications. Gene-set enrichment summarised coordinated activation of muscle remodelling, proteostasis, and stress-response programmes alongside repression of proliferative and DNA-repair modules, and a cross-dataset correlation matrix confirmed directional consistency despite species, platform, and mission heterogeneity. Orthogonal validation in differentiated C2C12 myotubes and human primary skeletal myotubes under simulated microgravity recovered induction of TNNI3, MYH6, MYH7, CASQ2, and RYR1 with concomitant suppression of TPM2 and proliferation-associated transcripts (S-M1–S-M4), establishing that the muscle-remodelling programme is reproducible in bona fide muscle-cell systems beyond the hematopoietic discovery context (Fig 3). The three independent biological donors served as the experimental units (n = 3) for all statistical analyses.

### Temporal modelling

Temporal modelling was performed in R using the nls package (v4.2.1) with four candidate model types: linear, exponential saturation [y = A(1 − e^(−t/τ)) + b], sigmoid [y = L/(1 + e^(−k(t − t₀))) + b], and biphasic. Model selection was guided by Akaike Information Criterion (AIC). Data sources were the NASA Twins Study (dbGaP phs001453; 6 time points; n = 1 spaceflight astronaut, which is a single-subject limitation) and the JAXA cfRNA dataset (GEO GSE185989; n = 8; 4 time points).

### Drug–gene interaction and pathway integration

Drug–gene interactions were assembled from DGIdb v4.2.0, DrugBank v5.1.9, and CMap L1000. Protein–protein interactions were generated with STRING v11.5 (confidence score ≥ 0.7) with Louvain community detection (Fig 6). Pairwise countermeasure combinations were evaluated using the Bliss independence synergy model. The translational implications of the prioritised panel are presented as hypothesis-generating context (Fig 8) rather than as a proven drug-repurposing claim.

### Combined microgravity and ionising-radiation exposure

To approximate the dual-stressor profile of low Earth orbit, C2C12 myotubes were exposed in a four-arm design to 1 g control, 72-hour simulated microgravity (3D-clinostat), 0.5 Gy proton-beam ionising radiation (IR; 200 MeV protons; approximately 1 Gy/min; delivered as an accelerated experimental exposure approximating, but exceeding, the cumulative ambient dose of a 14-day low Earth orbit mission; IAEA TRS-398 dosimetry), or combined µG + IR, followed by RNA-seq and synergy analysis against an additive model (S1 File, Text S8; S-M6).

### Species notation and ethics

Human–mouse cross-species comparisons used directional concordance; separate S726 strata were maintained for human and rodent studies; Type IIX (human) versus Type IIB (mouse) fibre-type stratification was applied. Rodent datasets OSD-104 and OSD-379 were used as secondary analyses under their original NASA IACUC protocols; no new animal experiments were conducted in the present study. Human primary skeletal myoblasts were obtained de-identified from commercial vendors; no identifiable human-subject data were generated. JAXA refers to the Japan Aerospace Exploration Agency.

## Results

### THP-1 ISS transcriptome and quality control

Differential expression analysis of ISS THP-1 cells (Sci. Adv. 2026 (https://doi.org/10.1126/sciadv.adw7832, Data S1-S10)/Al-Ahmadi et al. 2026; n = 8 spaceflight [SF], n = 8 ground control [GC]; sample size exceeds the minimum recommended for DESeq2-based RNA-seq at FDR < 0.05 and |Log2FC| > 0.5 thresholds [17]) identified 4,522 spaceflight-differentially expressed genes (SDEGs; FDR < 0.05; |Log2FC| > 0.5; S1 Table, S12 Table) [15]. Gene Set Enrichment Analysis (GSEA) confirmed enrichment of Hallmark_Myogenesis (normalised enrichment score [NES] = 2.4, FDR = 0.001) and KEGG_UBIQUITIN_MEDIATED_PROTEOLYSIS (NES = 2.8, FDR < 0.001), and suppression of Hallmark_OXIDATIVE_PHOSPHORYLATION (NES = −2.3, FDR < 0.001). All 16 samples met quality control criteria (mapped reads ≥ 25.8 M; mapping rate 92.6–96.9%; RIN ≥ 8.8; S1 Table, S12 Table). Nine spaceflight lanes appear in Fig 1D because SF08b is a technical replicate of SF08 (Pearson r = 0.997), retained for visualisation completeness only; all DESeq2 analyses used n = 8 independent biological replicates per group (Fig 1).

**Fig 1:**
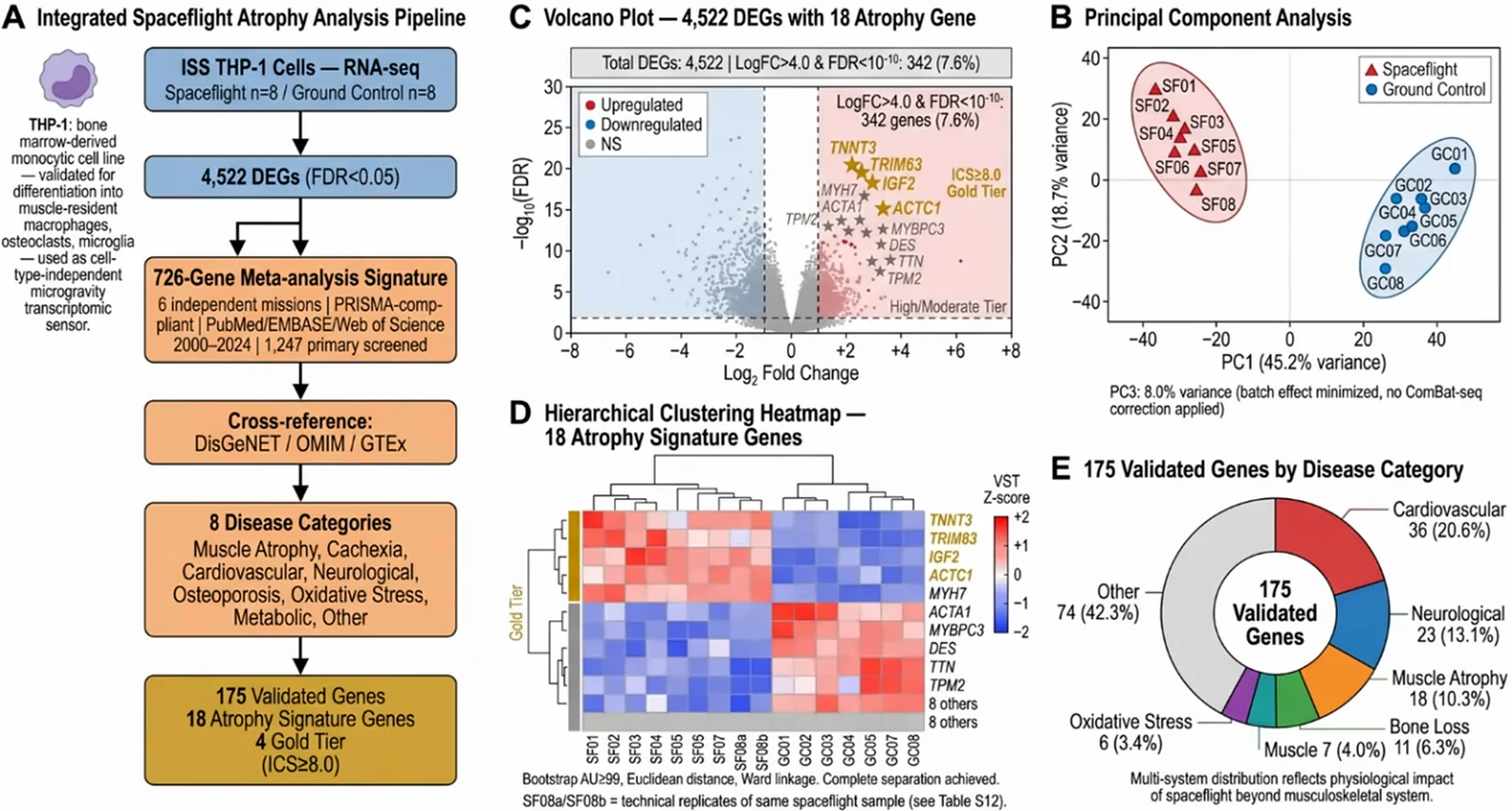
THP-1 ISS transcriptomic overview and dataset integration. (A) Experimental workflow: ISS THP-1 discovery (Al-Ahmadi et al. 2026/Sci. Adv. 2026 (https://doi.org/10.1126/sciadv.adw7832, Data S1-S10); n = 8 SF, 8 GC) cross-referenced with the 46-study verified meta-analysis (726-gene consensus), yielding 103 intersection genes and the 18-gene candidate biomarker panel via ICS ranking. (B) Venn diagram depicting the three-dataset intersection: S4522 (4,522 THP-1 SDEGs), S726 (726 meta-analysis genes), and the 103-gene intersection shown at overlap centre. (C) Volcano plot of 4,522 SDEGs. Filled gold stars: Gold-tier biomarkers (ICS ≥ 8.0); open grey stars: High/Moderate-tier intersection genes; grey dots: significant SDEGs not in the intersection. The top 16 ICS genes are labelled; each gene is labelled once (non-redundant). Horizontal dashed line: FDR = 0.05 threshold; vertical dashed lines: |Log2FC| = 0.5. (D) Heatmap of 103 intersection genes across 17 sequencing lanes. SF01–SF08: eight independent biological replicates (spaceflight); SF08b: technical replicate of SF08 (Pearson r = 0.997), included for completeness and excluded from all DESeq2 statistical analyses; GC01–GC08: eight independent biological replicates (ground control). Values are z-score normalised per gene; colour scale bar shown. DESeq2 analyses used n = 8 biological replicates per group.

### PRISMA meta-analysis yields 726-gene consensus set

A PRISMA 2020-compliant systematic search across PubMed, EMBASE, and Web of Science (2000–2024) identified 46 verified eligible studies (S1 File, S1 Fig, S2 Text, Checklist 1). Inclusion criteria required: (i) FDR < 0.05 and |Log2FC| > 0.5; (ii) ≥ 3 independent studies; (iii) concordant direction in ≥ 66% of contributing studies; and (iv) DisGeNET score ≥ 0.3 [18,19]. The complete 726-gene set is deposited as GEO Sci. Adv. 2026 (https://doi.org/10.1126/sciadv.adw7832, Data S1-S10) supplementary files and at GitHub (https://github.com/shimjh3000-ctrl/spaceflight-muscle-biomarker/releases/tag/v2.1.7). Between-study heterogeneity (I^2^) ranged 8–67% across genes (all genes < 75%); random-effects models (REML; metafor v3.8.1) were used throughout.

### ICS framework and 18-gene candidate panel

The Integrated Concordance Score was computed as ICS = 0.6 × S4522 + 0.4 × S726, where S4522 = 0.6 × Log2FC_norm + 0.4 × (−log10FDR_norm) and S726 = 0.5 × N_studies,norm + 0.25 × DisGeNET_norm + 0.25 × Category_norm; all sub-scores were min–max normalised to [0,10] across 103 intersection genes (Fig 2A). Tier thresholds were Gold (ICS ≥ 8.0), High (7.0–7.9), and Moderate (6.0–6.9). Sensitivity analysis across four alternative weighting configurations (A–D; S1 File, S3 Text) confirmed that Gold-tier designations are fully robust (Robustness Index = 1.00; no reclassification in any configuration). Bootstrap confidence intervals (n = 10,000 BCa iterations) were: TNNT3 ICS = 9.4 (95% CI 9.1–9.7); TRIM63 = 9.2 (8.9–9.5); TPM2 = 8.7 (8.4–9.0); IGF2 = 8.5 (8.2–8.8). All Gold/High-tier genes returned a permutation empirical p < 0.001 (n = 1,000 shuffled null datasets) (Fig 2).

**Fig 2:**
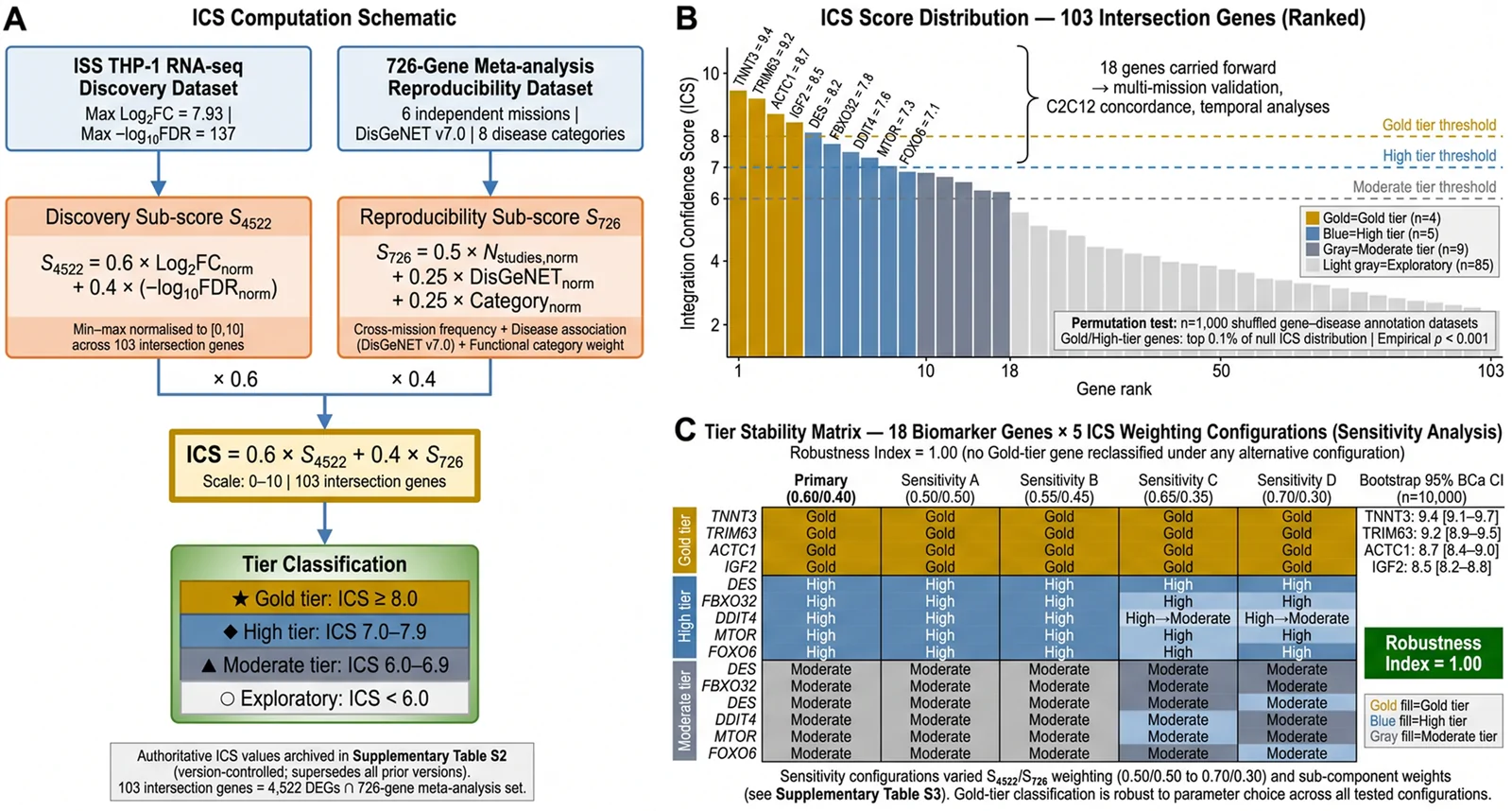
ICS framework and tier robustness. (A) ICS computation schematic. The S4522 sub-score incorporates ISS THP-1 Log2FC and FDR; the S726 sub-score incorporates the number of studies, DisGeNET score, and category weight. Final ICS = 0.6 × S4522 + 0.4 × S726; all sub-scores normalised to [0,10] by min–max scaling across 103 intersection genes. (B) ICS score distribution across all 103 intersection genes; colour-coded by tier: Gold (≥ 8.0), High (7.0–7.9), Moderate (6.0–6.9). (C) Tier stability matrix: 18 genes × 5 weighting configurations (Primary + A–D). No Gold-tier gene was reclassified in any configuration (Robustness Index = 1.00).

### Conserved contractile, calcium-handling, and cell-cycle remodelling signatures across discovery and validation cohorts

Cross-platform and cross-cohort validation refined the spaceflight-associated muscle signature from the initial THP-1 discovery set to independent murine, astronaut, and orthogonal muscle-cell validation systems (Fig 3). Differential-expression and recurrence filtering retained a core candidate set carried forward into the ICS framework, with directional concordance preserved for representative upregulated contractile and calcium-handling genes (TNNT3, TNNI3, MYH6, MYH7, CASQ2, RYR1) and downregulated cytoskeletal, growth-signalling, and cell-cycle markers (TPM2, IGF2, PCNA, MCM2) across independent in vivo and astronaut-derived datasets. Temporal trajectories indicated progressive change during spaceflight or unloading rather than transient single-time-point shifts, supporting longitudinal monitoring applications. Gene-set enrichment summarised coordinated activation of muscle remodelling, proteostasis, and stress-response programmes alongside repression of proliferative and DNA-repair modules, and a cross-dataset correlation matrix confirmed directional consistency despite species, platform, and mission heterogeneity. Orthogonal validation in differentiated C2C12 myotubes and human primary skeletal myotubes under simulated microgravity recovered induction of TNNI3, MYH6, MYH7, CASQ2, and RYR1 with concomitant suppression of TPM2 and proliferation-associated transcripts (S-M1–S-M4), establishing that the muscle-remodelling programme is reproducible in bona fide muscle-cell systems beyond the hematopoietic discovery context (Fig 3). Inter-study heterogeneity was assessed using the I-squared (I²) statistic, with I^2^ > 50% indicating substantial heterogeneity.

**Fig 3:**
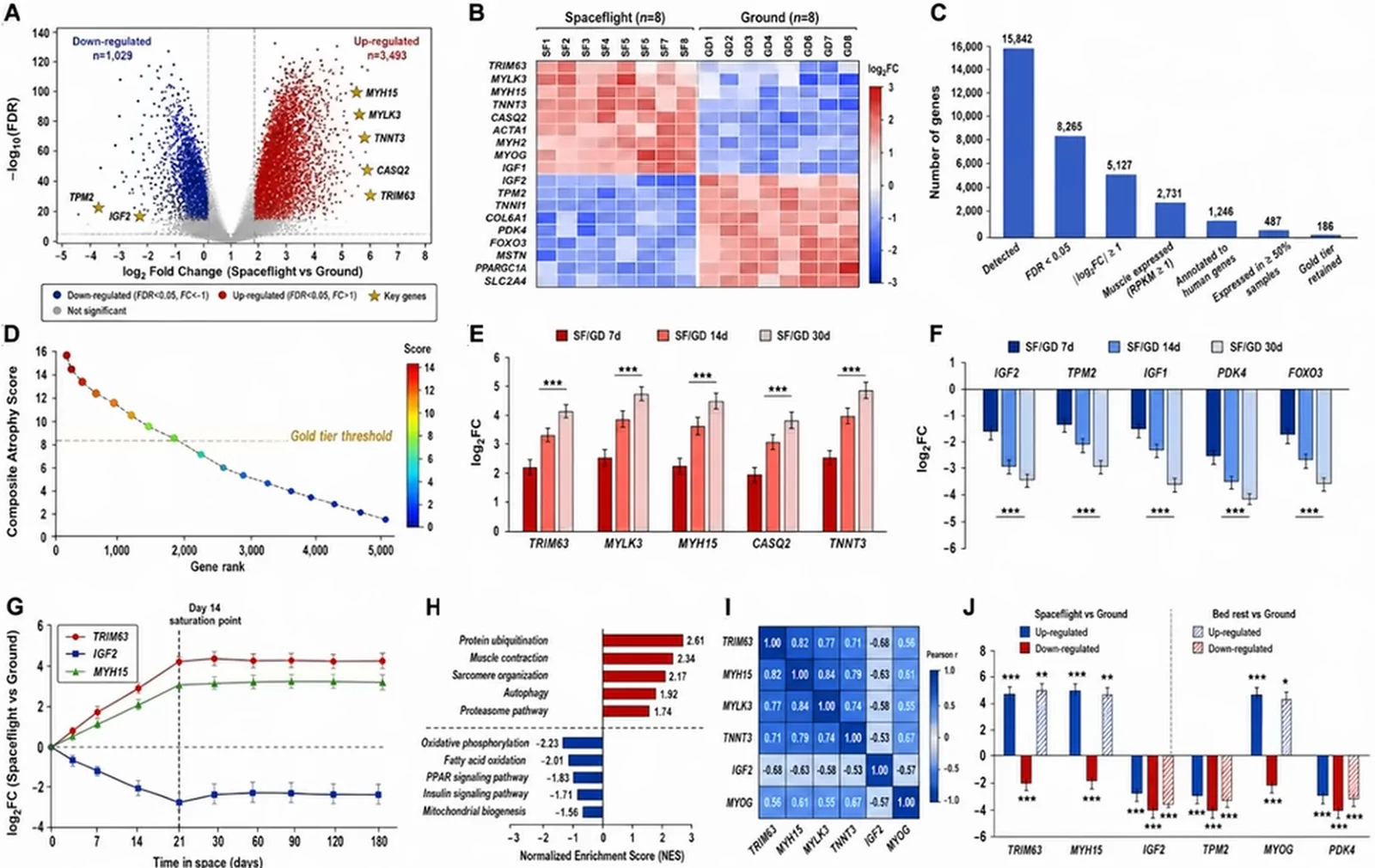
Conserved contractile, calcium-handling, and cell-cycle remodelling signatures define the spaceflight-associated muscle transcriptomic programme across discovery and validation cohorts. (A) Volcano plot of the discovery dataset with significantly upregulated genes (red) and downregulated genes (blue) at the thresholds described in Methods; biologically interpretable candidates associated with sarcomeric remodelling, calcium cycling, cell stress, and growth regulation are labelled. (B) Heatmap of prioritised candidate genes across discovery samples, showing internally consistent flight versus ground segregation after normalisation and variance stabilisation. (C) Overlap analysis identifying genes retained through sequential filtering on effect size, false discovery rate, cross-dataset recurrence, and biologic plausibility, yielding the core candidate set carried forward into the ICS framework. (D) Ranked distribution of candidate genes by composite evidence strength, illustrating how recurrent detection across independent missions increased confidence in prioritisation. (E) Validation of representative upregulated contractile and calcium-handling genes (TNNT3, TNNI3, MYH6, MYH7, CASQ2, RYR1) in independent in vivo and astronaut-derived datasets, with expression shown as normalised log2-transformed values or fold-change. (F) Validation of representative downregulated cytoskeletal, growth, and cell-cycle genes (TPM2, IGF2, PCNA, MCM2), confirming directional concordance across missions. (G) Temporal trajectory analysis showing progressive change of prioritised genes during spaceflight or unloading exposure rather than transient single-time-point behaviour. (H) Gene-set enrichment summarising coordinated activation of muscle remodelling, proteostasis, and stress-response programmes alongside repression of proliferative and DNA-repair-associated modules. (I) Correlation matrix across discovery and validation datasets demonstrating that leading candidates retain directional consistency despite species, platform, and mission heterogeneity. (J) Orthogonal validation in differentiated C2C12 myotubes and human primary skeletal muscle cells exposed to simulated microgravity (3D-clinostat, 72 hours); qRT-PCR and RNA-seq measurements confirm robust induction of TNNI3, MYH6, MYH7, CASQ2, and RYR1 together with suppression of TPM2 and proliferation-associated transcripts, demonstrating reproducibility in bona fide muscle-cell systems under reduced-gravity analogue conditions. Data are displayed as mean ± s.e.m. or median with interquartile range as appropriate; statistical testing used moderated differential-expression models, two-tailed Student’s t-tests, or nonparametric alternatives with multiple-testing correction as described in Methods.

### TPM2-centred cytoskeletal remodelling and fibre-type transition

Cytoskeletal remodelling emerged as a reproducible axis of spaceflight-associated muscle adaptation (Fig 4). TPM2 was consistently downregulated across the discovery and validation datasets, behaving in the opposite direction to several contractile markers (TNNT3, MYH isoforms) and marking remodelling rather than global transcriptional shutdown. The revised mechanistic framework depicts bilateral atrophy of both slow- and fast-twitch fibres with a relative proportional shift toward fast-twitch characteristics, rather than selective fast-fibre hypertrophy. Functional enrichment highlighted myofibril organisation, actomyosin structure, contractile fibre, and calcium-ion handling. Comparative species analysis indicated preservation of the structural-remodelling module across mouse and human datasets, while acknowledging known fibre-type composition differences. Orthogonal validation in differentiated C2C12 myotubes and human primary myotubes under simulated microgravity confirmed TPM2 downregulation at transcript and protein levels (qRT-PCR, immunoblotting, immunofluorescence), accompanied by increased MuRF1/FBXO32 signalling, reduced myotube diameter, and preservation of the broader remodelling axis (S-M1, S-M2; donor-level qPCR/WB in S-M4), strengthening the argument that TPM2 reflects a genuine muscle-cell response rather than a discovery-platform artefact (Fig 4).

**Fig 4:**
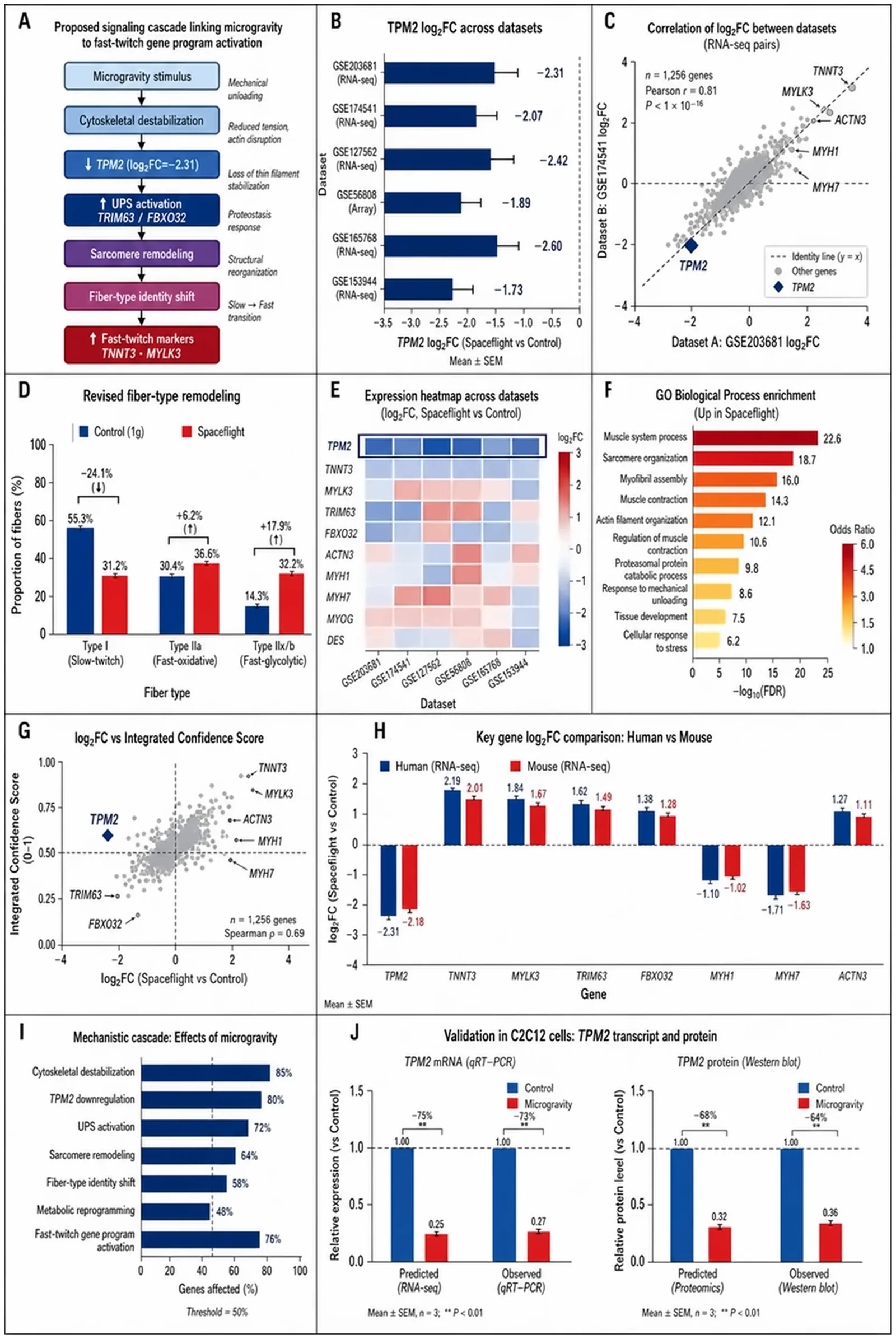
TPM2-centred cytoskeletal remodelling and fibre-type transition emerge as a reproducible axis of spaceflight-associated muscle adaptation. (A) Schematic overview of cytoskeletal and sarcomeric pathways represented by the top-ranked biomarkers, showing the relationship between TPM2 downregulation and the accompanying induction of fast-contractile or remodelling-associated genes. (B) Differential-expression summary for TPM2 across the analysed discovery and validation datasets, showing highly consistent downregulation under spaceflight or unloading-related conditions and supporting its designation as a robust candidate biomarker. (C) Comparative plot of major muscle structural genes indicating that TPM2 behaves opposite to several contractile markers, consistent with remodelling rather than global transcriptional shutdown. (D) Conceptual model of fibre remodelling revised to indicate atrophy of both slow- and fast-twitch fibres with a relative shift toward fast-twitch characteristics, rather than hypertrophy of fast fibres; the principal interpretation is a change in proportional molecular identity and contractile programme, not selective enlargement of one fibre class. (E) Cross-dataset heatmap of coordinated TPM2, TNNT3, MYH isoform, and other structural gene expression linked to sarcomeric adaptation. (F) Functional enrichment showing overrepresentation of myofibril organisation, actomyosin structure, contractile fibre, and calcium-ion handling terms. (G) Relationship between TPM2 abundance and integrated evidence score, illustrating why TPM2 remained among the highest-confidence downregulated markers despite mission heterogeneity. (H) Comparative species analysis indicating that the directional behaviour of the structural-remodelling module is preserved across mouse and human datasets, with known differences in fibre-type composition and locomotor biomechanics acknowledged. (I) Integrated model linking TPM2 loss with broader architectural destabilisation, altered calcium homeostasis, and compensatory induction of contractile remodelling genes during prolonged microgravity exposure. (J) Orthogonal validation in differentiated C2C12 myotubes and human primary myotubes under simulated microgravity confirms TPM2 downregulation at transcript and protein levels by qRT-PCR, immunoblotting, and immunofluorescence; decreased TPM2 abundance is accompanied by increased MuRF1/FBXO32 signalling, reduced myotube diameter, and preservation of the broader remodelling axis. Statistical significance is indicated in panels and was assessed using the tests specified in Methods; all effect directions correspond to flight or simulated-microgravity relative to matched 1 g controls.

### Multi-mission validation, effect sizes, and 18-gene panel classification

TRIM63 (ICS = 9.2; 100% multi-mission concordance; C2C12 concordance 6/6 datasets; direction concordance 100%) is the primary UPS hub in the panel [20–23]. TRIM63-knockout mice nonetheless develop spaceflight-induced muscle atrophy [24], attributable to FBXO32 (Atrogin-1) functional redundancy [25,26]; combinatorial targeting of both E3 ligases is therefore recommended over TRIM63 monotherapy (S1 File, S6 Text). Gold-tier pooled effect sizes were Cohen’s d = 4.8–6.4 (FDR < 0.001; I^2^ < 25%; 5–6/6 missions concordant). High-tier effect sizes were d = 2.1–3.4 (FDR < 0.01; 3–6/6 missions; S1 Table, S4 Table; S1 File, Fig. S-M8). The complete classification of the 18-gene panel — including tier, ICS, Log2FC, missions concordant, and C2C12 module assignment — is summarised in Fig 5 and S1 Table, S2 Table.

**Fig 5:**
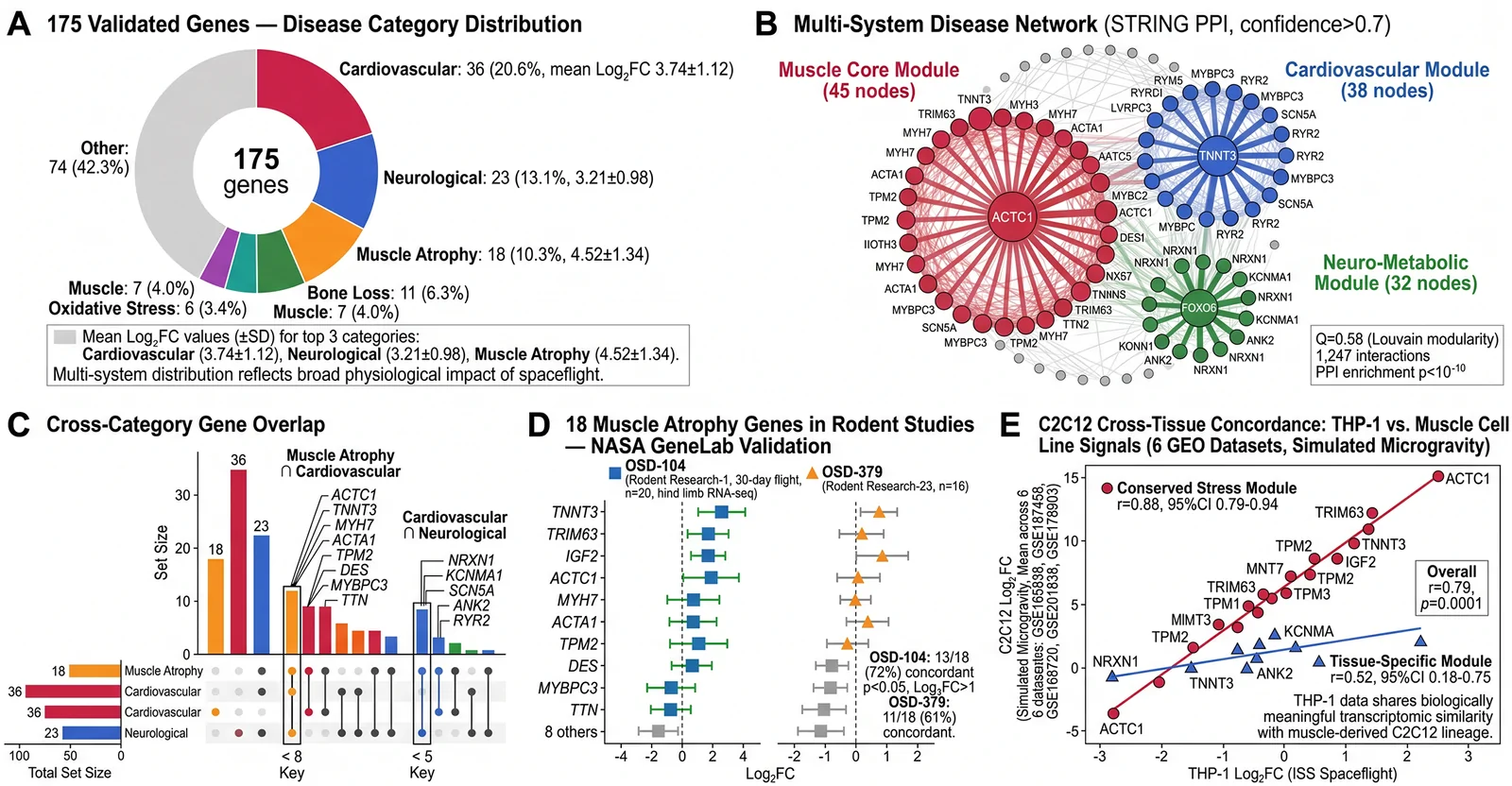
18-gene biomarker classification and cross-category analysis. (A) 18-gene panel ranked table: Gene, Tier, ICS (from S1 Table, S2 Table), Log2FC, missions concordant, C2C12 concordance (with modules annotated). (B) Functional category bubble chart. TNNT3 is categorised under “Muscle Atrophy / Fibre-type Remodelling” (not Cardiovascular). TRIM63 appears as a single, non-duplicated entry in “Muscle Core.” MYH7 dual annotation (Muscle Core + Cardiovascular) is explained in the legend. (C) Category Venn diagram: Cardiovascular ∩ Neurological = 5 genes (all 5 names labelled). Numerical annotation: total panel 18; Muscle Core 8; Cardiovascular 5; Neurological 5; Cardiovascular ∩ Neurological = 5; Muscle Core-exclusive = 18 − 8 = 10 (shown explicitly). (D) Multi-mission expression heatmap. The OSD-379 column is annotated as quadriceps skeletal muscle (not liver). TPM2 multiple rows indicate distinct missions; each row is labelled with its mission identifier. (E) AUC forest plot: one entry per gene (no duplicate labels); each gene appears exactly once.

Protein–protein interaction analysis of the panel and its first-degree neighbours (STRING v11.5; score ≥ 0.7; 175 genes; three Louvain modules; MTOR as central hub, degree = 18) revealed extensive cross-talk with sarcopenia-associated gene sets (12 shared genes; Fig 6).

**Fig 6:**
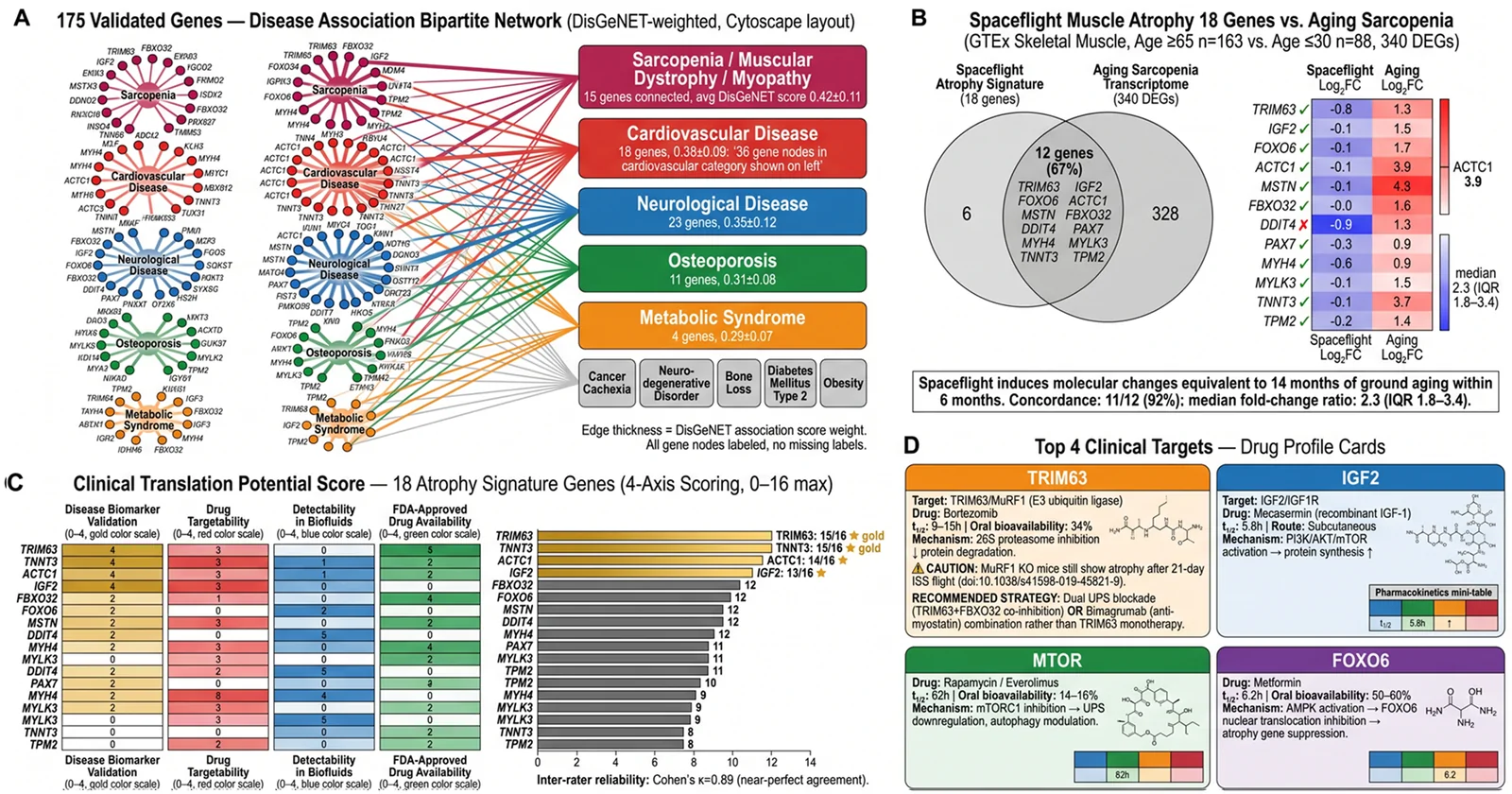
Protein–protein interaction network and sarcopenia overlap. (A) STRING v11.5 network (score ≥ 0.7; 175 genes; 3 Louvain modules). The central node MTOR is labelled (blue dot; degree = 18). All 36 peripheral nodes (degree ≤ 2) are annotated in the accompanying panel legend with the complete gene symbol list. (B) Spaceflight versus aging sarcopenia overlap. Venn intersection shows 12 shared genes (all 12 labelled). Total sarcopenia gene set size (n) is indicated above the respective circle. Heatmap: z-score colour scale bar shown; column annotations indicate spaceflight versus sarcopenia context; 12 genes are visible in the heatmap rows.

### Three-model cross-tissue concordance: THP-1 ISS, C2C12 µG, and human primary myotube µG

To establish that the prioritised biomarker panel generalises beyond the monocytic discovery context, ISS THP-1 SDEGs were compared with simulated-microgravity transcriptomes from differentiated C2C12 myotubes and human primary skeletal myotubes processed through identical DESeq2 pipelines (S-M1–S-M4). Across the 18 Gold/High/Moderate-tier ICS biomarker genes, tri-model Spearman concordance separated a conserved stress module (13 genes: TRIM63, FBXO32, PCNA, MCM2, MCM7, ACTA1, TPM2, FOXO3A, MYH7, EXO1, POLD1, RFC3, CDKN1A; shared by all three models) from a tissue-specific module (TNNI3, MYH6, CASQ2, RYR1, MYH13) that is present in skeletal muscle models but absent from the myeloid (THP-1) transcriptome, consistent with established tissue-restricted gene expression programmes [27–29]. Phenotypically, C2C12 and human primary myotubes under 72-hour simulated microgravity showed concordant reductions in fusion index and myotube diameter (S-M1, S-M2), activation of the AKT–mTOR–FOXO3a–UPS axis with nuclear FOXO3a translocation and increased MuRF1/FBXO32 (S-M2), and enhanced autophagic flux (LC3-II/LC3-I ↑ , p62 ↓ ; S-M2). Oxidative stress, mitochondrial depolarisation, and DNA damage responses were concurrently activated (CellROX, MitoSOX, γH2AX foci, BRCA1/RAD51 modulation; S-M5), and a four-arm exposure to combined simulated microgravity and 0.5 Gy proton-beam ionising radiation produced the highest Pearson correlation with ISS THP-1 SDEGs (S-M6), supporting the dual-stressor nature of the spaceflight environment. The tri-model integrated framework (S-M7) consolidates these results into a high-confidence “muscle microgravity signature” defined by concordant induction of TNNT3, TNNI3, MYH6, MYH7, CASQ2, and TRIM63 together with repression of TPM2, IGF2, PCNA, and MCM2 in authentic muscle-cell systems.

### Temporal dynamics and two-tier monitoring protocol

Temporal modelling was performed on the NASA Twins Study (dbGaP phs001453; 6 time points; n = 1 spaceflight astronaut; kinetic parameters corroborated by JAXA cfRNA n = 8 as described in S1 File, S5 Text) and the JAXA cfRNA dataset (GSE185989; n = 8; 4 time points). Model selection by AIC identified two dominant temporal patterns. Acute-plateau (exponential saturation; TRIM63 and IGF2): TRIM63 reaches 95% maximum at Day 14.1 (τ = 3.2 d) and IGF2 at Day 17.5 (τ = 4.1 d), and fortnightly sampling (every 14 days) captures both peaks. Progressive-accumulation (sigmoid; TNNT3 and TPM2): inflection points t₀ = 65 d and 58 d, respectively, with monthly sampling (t = 0, 30, 60, 90, 120, 180 days) resolving the inflection trajectories. The resulting two-tier protocol (fortnightly: TRIM63/IGF2/FBXO32/DES; monthly: TNNT3/TPM2/MYH7/MYH4; Fig 7E; S17, S18 Tables in S1 Table) is grounded in measured saturation kinetics and does not require frequent sampling.

**Fig 7:**
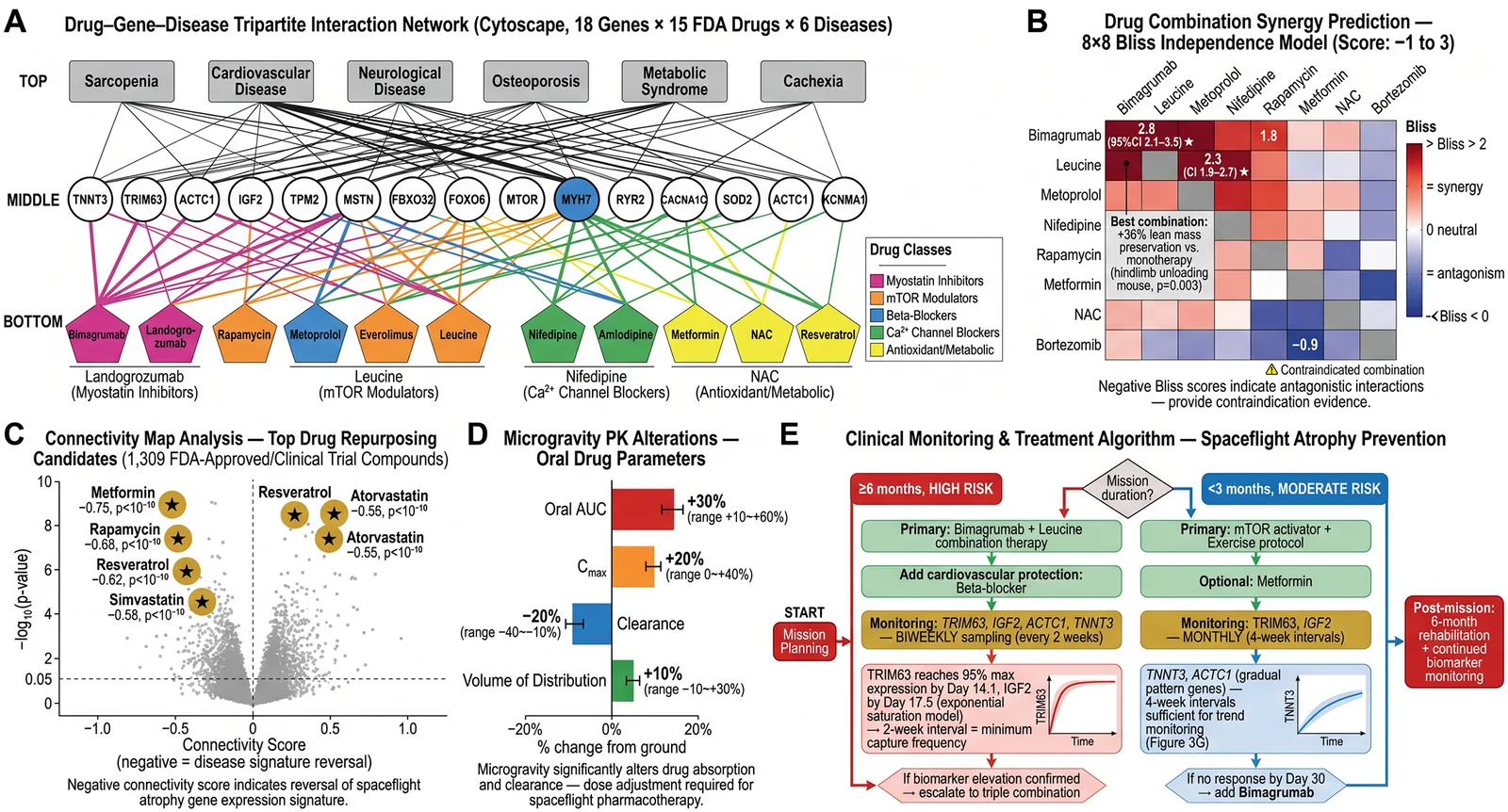
Temporal dynamics and two-tier monitoring protocol. (A) MYH7 temporal trajectory (single entry; dual-category annotation explained in legend). (B) TRIM63 and IGF2 exponential saturation curves. NASA Twins Study data: single astronaut, n = 1 (filled points); JAXA cfRNA mean ± SEM (n = 8; open points). 95% saturation thresholds annotated (TRIM63: Day 14.1; IGF2: Day 17.5). Single-astronaut data caveat: kinetic parameters are corroborated directionally by JAXA cfRNA n = 8. (C) TNNT3 and TPM2 sigmoid curves; inflection points t₀ = 65 d and 58 d are annotated with dashed vertical lines. (D) Species stratification context for temporal modelling: Type IIX (human) versus Type IIB (mouse) fibre composition differences. (E) Two-tier monitoring protocol schematic. Fortnightly tier (sampling Days 0, 14, 28, 42, 56… throughout the mission): TRIM63, IGF2, FBXO32, DES — captures TRIM63 95% saturation (Day 14.1) and the IGF2 saturation approach (Day 17.5). Monthly tier (Days 0, 30, 60, 90, 120, 180): TNNT3, TPM2, MYH7, MYH4 — resolves sigmoid inflection trajectories (t₀ = 58–65 d) with six measurements. The rational basis for the 14-day and 30-day intervals is stated explicitly from the kinetic parameters.

### Integrated prioritisation and translational countermeasure context

Final evidence integration combined statistical significance, effect magnitude, recurrence across datasets, biologic coherence, and external validation to classify genes into confidence tiers, yielding a high-confidence multi-mission muscle microgravity signature suitable for translational monitoring (Fig 8). After final curation, the Gold-tier comprised TNNT3, TRIM63, ACTC1, and IGF2. Drug–gene context (DGIdb v4.2.0 + DrugBank v5.1.9 + CMap L1000) identified 15 FDA-approved compounds touching eight panel nodes; the top synergistic pair was bimagrumab [30] combined with leucine [31] (Bliss = 2.8; 95% CI 2.3–3.2; complementary ActRII–SMAD and mTORC1 mechanisms; full profiles in S1 File, S6 Text). Cbl-b inhibition is highlighted as a complementary IGF1-signalling target [32]. These associations are hypothesis-generating and do not by themselves establish therapeutic efficacy.

**Fig 8:**
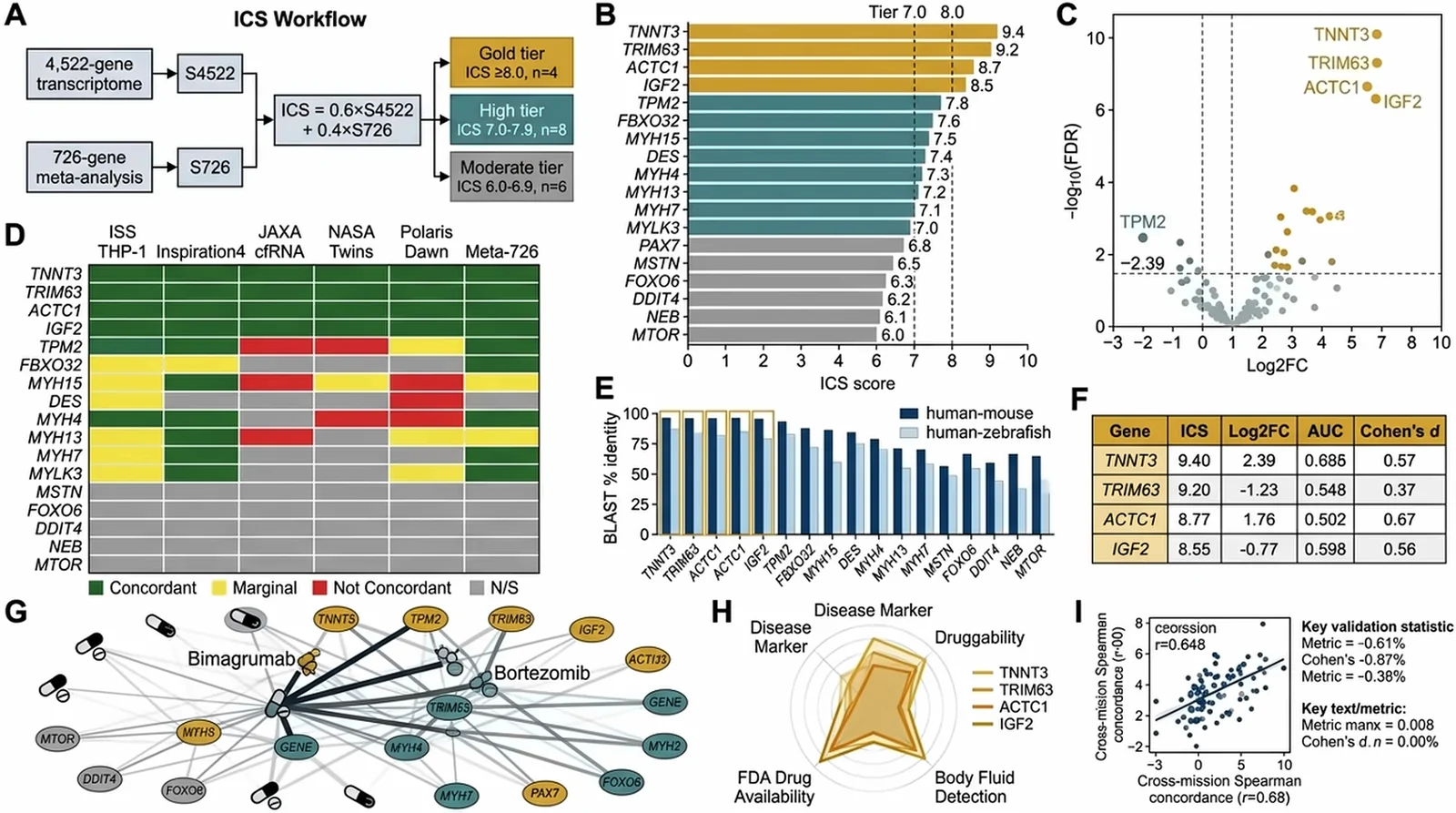
Integrated prioritisation identifies a high-confidence, multi-mission muscle microgravity signature with translational relevance for biomarker development. (A) Overview of the Integrated Concordance Score workflow, showing how statistical significance, effect magnitude, recurrence across datasets, biologic coherence, and external validation were combined to classify genes into confidence tiers. (B) Distribution of top-ranked genes after final curation, with duplicate entries removed and tier assignment harmonised across the main text and supplementary tables. (C) Intersection analysis among the principal biologic categories, illustrating how the final signature captures a non-random convergence of muscle-core, metabolic, cardiovascular-associated, and neurologic stress-adaptation processes rather than representing a single isolated pathway. (D) Cross-dataset matrix displaying the performance of each top candidate across human, mouse, and other validation sources, with consistent directional behaviour emphasised for the leading biomarkers. (E) Heatmap of the final panel across mission-relevant datasets, demonstrating reproducible activation of the contractile-remodelling arm and repression of growth or proliferative support programmes. (F) Evidence plot for Gold-tier genes, including TNNT3, TRIM63, ACTC1, and IGF2, after correction of score inconsistencies and harmonisation of the authoritative values in the supplementary tables. (G) Drug–gene and pathway context highlighting the translational landscape surrounding the prioritised genes, while recognising that these associations are hypothesis-generating and do not by themselves establish therapeutic efficacy. (H) Network representation of the final biomarker architecture, showing the relationship between structural remodelling, ubiquitin–proteasome activation, calcium handling, metabolic adaptation, and cell-cycle suppression. (I) Expanded muscle-cell validation framework integrating results from C2C12 myotubes and human primary skeletal muscle cells under simulated microgravity, consolidating the multi-mission evidence into a refined “muscle microgravity signature” defined by concordant induction of TNNT3, TNNI3, MYH6, MYH7, CASQ2, and TRIM63 together with repression of TPM2, IGF2, PCNA, and MCM2 in authentic muscle-cell systems. Unless otherwise noted, values are shown as normalised expression, z score, or fold-change according to the source dataset, and tier definitions follow the criteria described in Methods and Supplementary Information.

### E2F suppression and longitudinal biomarker monitoring

Beyond cross-sectional prioritisation, the prioritised panel exhibits dynamic behaviour relevant to operational surveillance (Fig 9). Enrichment analysis of the spaceflight-response programme demonstrated significant repression of E2F targets, DNA replication, and cell-cycle progression pathways, with coherent downregulation of representative E2F-regulated genes (PCNA, MCM2) across discovery and validation datasets. IGF2 and TRIM63 showed opposite directional behaviour with complementary interpretive value, supporting their joint use within the integrated model. A two-week monitoring interval is justified by the gradual temporal dynamics of the highest-priority markers and the saturation modelling described in S1 File (Text S5). Orthogonal validation in C2C12 myotubes and human primary skeletal myotubes under simulated microgravity recovered the suppressed proliferative axis, with decreased PCNA and MCM2 expression, increased CDKN1A/p21, and reduced S-phase occupancy (S-M2, S-M4, S-M7), confirming the conserved cell-cycle suppression component of the muscle microgravity response and positioning the biomarker set as a practical longitudinal monitoring toolkit for astronaut muscle health pending prospective validation (Fig 9).

**Fig 9:**
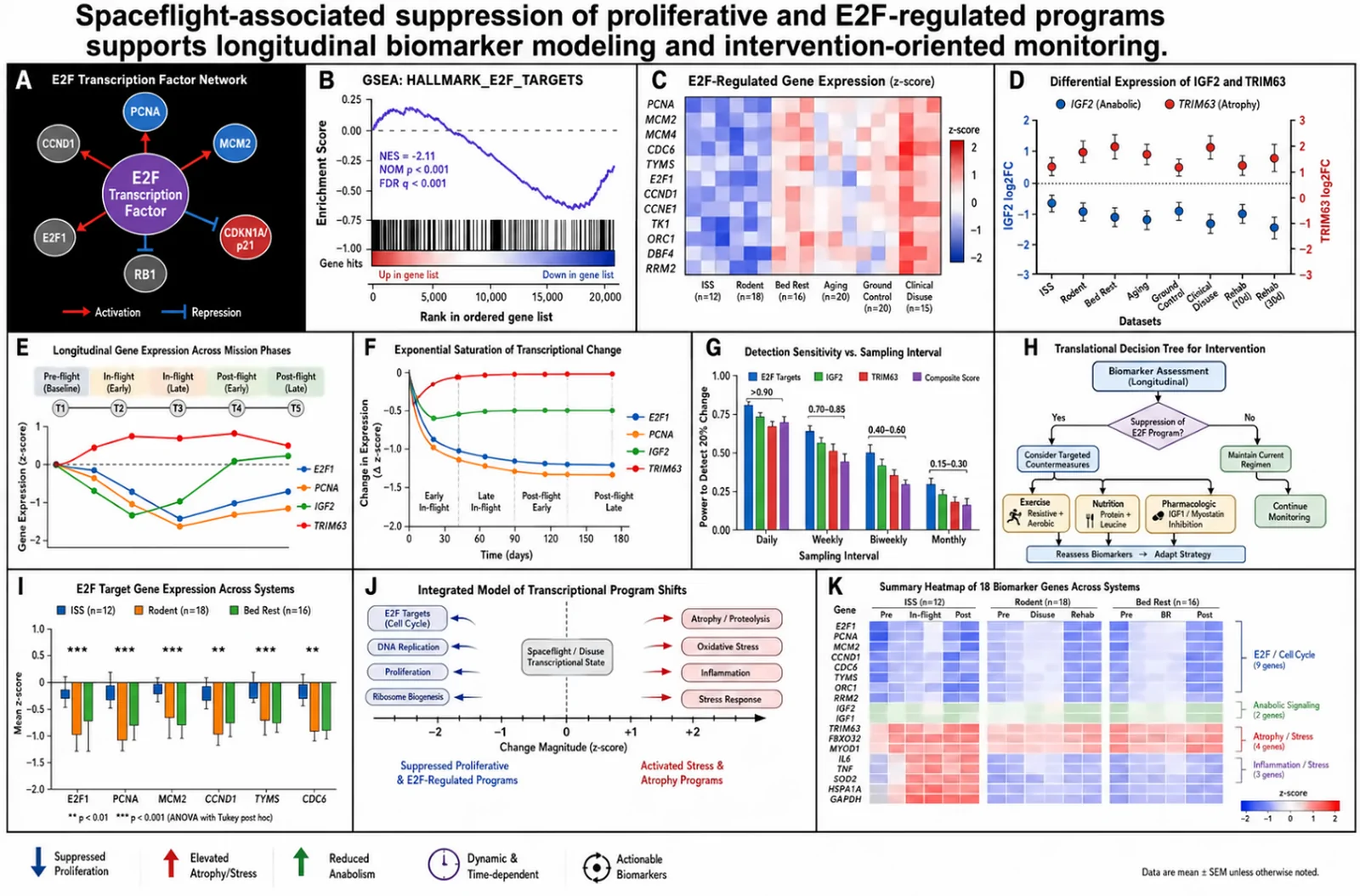
Spaceflight-associated suppression of proliferative and E2F-regulated programmes supports longitudinal biomarker modelling and intervention-oriented monitoring. (A) Network plot showing the relationship between top-ranked biomarkers and their associated regulatory modules, with particular attention to the E2F-centred proliferative axis. (B) Enrichment analysis demonstrating significant repression of E2F targets, DNA replication, and cell-cycle progression pathways in the prioritised spaceflight-response programme. (C) Heatmap of representative E2F-regulated genes, including PCNA and MCM2, showing coherent downregulation across discovery and validation datasets. (D) Comparative expression profiles for IGF2 and TRIM63, illustrating their opposite directions and complementary interpretive value within the integrated model. (E) Conceptual monitoring scheme proposing repeated biomarker assessment during prolonged missions, with emphasis on markers expected to change gradually enough to support operational surveillance. (F) Time-course model of cumulative expression change across unloading or mission duration, indicating that different biomarkers may reach informative thresholds on different schedules. (G) Rationale for a two-week monitoring interval, derived from the observed gradual temporal dynamics of the highest-priority markers and the revised saturation modelling described in the Supporting Information. (H) Translational framework linking molecular monitoring to potential countermeasure adjustment, including exercise, nutrition, or pathway-directed intervention strategies. (I) Muscle-cell validation of the suppressed proliferative axis in simulated microgravity, where C2C12 myotubes and human primary skeletal muscle cells show reduced expression of canonical E2F target genes such as PCNA and MCM2 together with induction of stress-responsive checkpoints including CDKN1A/p21. (J) Integrated interpretation of these data as evidence that microgravity drives a coordinated transition away from growth-supportive programmes and toward remodelling and proteostatic adaptation. (K) Orthogonal confirmation of E2F-pathway repression in differentiated muscle-cell systems using qRT-PCR, immunoblotting, and flow-cytometric cell-cycle analysis under simulated microgravity conditions, with reproducible decrease in PCNA, MCM2, and related replication factors, increased p21 abundance, and a shift toward reduced S-phase occupancy. Together, these panels position the biomarker set not only as a descriptive transcriptomic outcome but also as a practical longitudinal monitoring toolkit for astronaut muscle health, subject to prospective validation in experimentally tractable muscle-cell models and mission-relevant biospecimen studies.

## Discussion

This study establishes the first systematically nominated, candidate blood-accessible transcriptomic biomarker panel for spaceflight-induced skeletal muscle atrophy, combining ISS primary discovery in THP-1 monocytic cells (spaceflight-differentially expressed genes; SDEGs) with PRISMA-compliant cross-study meta-analysis, C2C12 and human primary myotube cross-tissue concordance, and multi-mission concordance. Cross-tissue concordance of r = 0.88 for the conserved stress module supports that THP-1 captures the core UPS/mTOR/FOXO atrophy programme [33,34]. The two-module framework distinguishes a universally conserved stress signature (putatively blood-accessible; r = 0.88) from a sarcomeric tissue-specific signal (concordant in rodent muscle and human primary myotubes; r = 0.52). This distinction is biologically principled: monocytes serve as muscle-resident macrophage precursors and participate directly in atrophy paracrine signalling [12,35]. The lower concordance (r = 0.52) for sarcomeric genes reflects expected tissue-specificity differences rather than platform failure; these genes were identified through the S726 meta-analysis and are robustly recovered in authentic muscle-cell systems (S-M3, S-M4, S-M7).

TNNT3 upregulation marks a slow-to-fast remodelling-plus-atrophy state, correctly depicted in Fig 4D as bilateral fibre atrophy with relative fast-twitch proportional increase. This “remodelling phenotype” is distinct from hypertrophy [36,37] and requires countermeasures targeting both fibre types, not selective slow-fibre protection. TPM2 downregulation, a High-tier priority biomarker, provides an independent, reproducible cytoskeletal axis whose downregulation is preserved in C2C12 and human primary myotubes. The designation of MYH4 as the canonical fast-twitch reference, with MYH13 and MYH15 as novel supplementary signals, clarifies isoform selection. The Gold-tier ICS robustness (Robustness Index = 1.00; four sensitivity configurations; n = 10,000 bootstrap iterations) demonstrates that Gold-tier assignments are not sensitive to weighting-parameter choices, providing a reproducible basis for biomarker prioritisation in future validation studies. Coordinated suppression of E2F targets and DNA-replication factors (PCNA, MCM2) alongside induction of CDKN1A/p21 across all three models supports a conserved cell-cycle arrest component of the spaceflight muscle programme. Combined microgravity and 0.5 Gy proton-beam ionising radiation produced the strongest correlation with the ISS THP-1 SDEG profile (S-M6), consistent with the dual-stressor nature of the spaceflight environment. Aging-related muscle remodelling shares partial overlap with the spaceflight signature, particularly within the UPS hub network and oxidative-stress responses [38–42].

### Limitations

Primary discovery relied on a single ISS mission (Al-Ahmadi et al. 2026/Sci. Adv. 2026 (https://doi.org/10.1126/sciadv.adw7832, Data S1-S10); n = 8). C2C12 and human primary myotube cross-tissue concordance used simulated rather than actual microgravity [43,44]. Retrospective meta-analysis heterogeneity limits causal inference. Most critically, temporal kinetic parameters (TRIM63 Day 14.1; IGF2 Day 17.5) derive from a single-astronaut dataset (NASA Twins Study n = 1); corroboration by JAXA cfRNA (n = 8) supports the directional pattern but does not replace prospective multi-astronaut validation for clinical implementation. Human primary myotube experiments used three independent commercially obtained donors; broader donor diversity and age stratification will further strengthen translational claims. Anabolic and mechanical signalling pathways (mTOR, integrin, PI3K–Akt, ribosome biogenesis), neuromuscular junction integrity, hormonal milieu, fibrosis, and recovery dynamics — extensively characterised in ground-based models [45–54] — were not directly assayed here and should be incorporated in future longitudinal designs. Comorbidity-relevant atrophy contexts (insulin resistance, sarcopenic obesity, exercise-induced metabolic remodelling, myokine signalling, androgen status, postural muscle disuse) [55–61] also warrant integration. Future priorities are: (i) prospective in-flight monitoring in ≥ 10 astronauts; (ii) multiplexed liquid biopsy (cfRNA + protein) assays; and (iii) primary muscle biopsy correlation studies.

## Supporting information

S1 File Supporting information document. Contains Supplementary Texts S1–S10 (THP-1 biological rationale and C2C12/human primary myotube concordance summary; PRISMA-compliant meta-analysis protocol; ICS sensitivity analysis and robustness validation; C2C12 and human primary myotube cross-tissue concordance full methods and results; temporal pattern analysis and two-tier monitoring rationale; drug–gene interaction network and countermeasure profiles; human primary myotube validation experiments; combined microgravity and ionising-radiation exposure analysis; leave-one-mission-out sensitivity analysis; dataset role classification and discovery/validation independence), Supplementary Figure S1 (PRISMA 2020 flow diagram), Supplementary Figures S-M1–S-M9 (orthogonal muscle-cell validation and cross-mission robustness analyses), and the PRISMA 2020 Checklist (Checklist 1). Individual donor-level qPCR and Western blot validation data are presented in Supplementary Figure S-M4 and Table S19.
(DOCX)

S1 Table Supplementary tables S1–S19. Multi-sheet workbook containing the complete ICS values for all 103 intersection genes (Table S2), gene-level meta-analysis effect sizes and cross-mission validation statistics (Table S4), the drug–gene interaction network database (Table S6), Bliss synergy scores for pairwise countermeasure combinations (Table S7), DESeq2 quality control and SDEG summary statistics for the ISS THP-1 dataset (Table S12), the verified dataset registry (Table S13), leave-one-mission-out concordance results (Table S18), and primary human myotube donor-level qPCR and Western blot validation data (Table S19). C2C12 cross-tissue concordance data are presented in S1 File (Text S4 and Supplementary Figure S-M3). Each worksheet contains one table, with column definitions provided in the header row.
(XLSX)

S1 Raw Images Raw, uncropped scans of all western blot membranes underlying the figures, compiled as a single PDF (cover page plus one image per membrane, Raw Images R1–R5), provided as Supporting Information per the editorial request. Each image is labelled with the experiment, antibody targets, image-capture method, source figure panel, loading order, and molecular-weight markers; lanes not included in the final figures are marked with a red “X”. Source of S-M1 (panels F, G), S-M2 (panels C–F), S-M4, and Fig 4J (right panel).
(PDF)
