# Supplementary material for: Integrative transcriptomics identifies an 18-gene candidate blood-accessible biomarker panel for longitudinal monitoring of spaceflight-induced muscle atrophy
Source: PLoS One. 2026 Oct 7;21(10):e0358130. doi: 10.1371/journal.pone.0358130 (PMC13645052; doi:10.1371/journal.pone.0358130)

S1_raw_images

Uncropped original blot and gel images

Manuscript: PONE-D-26-24379R1

Title: Integrative transcriptomics identifies an 18-gene candidate blood-accessible biomarker panel for longitudinal monitoring of spaceflight-induced muscle atrophy

Date: 1 September 2026

Contents

Raw Image R1

C2C12 myotubes (1 g vs simulated microgravity, 72 h): MyHC, alpha-tubulin, MuRF1/TRIM63, MAFbx/FBXO32; three biological replicate membranes.
Source of: Supplementary Fig S-M1, panels F (representative blots) and G (densitometry)

Raw Image R2

C2C12 myotubes: p-AKT/total AKT, p-mTOR/total mTOR, p-FOXO3a/total FOXO3a, beta-actin (stripping/re-probing); three biological replicate membranes.
Source of: Supplementary Fig S-M2, panels C (representative blots) and D (densitometry)

Raw Image R3

C2C12 myotubes +/- bafilomycin A1 (100 nM, 4 h): LC3A/B, p62/SQSTM1, beta-actin; three biological replicate membranes.
Source of: Supplementary Fig S-M2, panels E (representative blots) and F (densitometry)

Raw Image R4

Primary human myotubes, 3 donors, 18-gene panel (TNNT3 ... CASQ2) + beta-actin; composite of uncropped membranes from raw scans R4-01 to R4-54.
Source of: Supplementary Fig S-M4 (donor-level qPCR and Western blot fold-change quantification)

Raw Image R5

C2C12 myotubes: TPM2 and beta-actin.
Source of: Fig 4J, right panel (TPM2 protein quantification by Western blot)

General notes

- All images in this file are uncropped original scans. No adjustment of brightness, contrast, or exposure was applied to any image; text banners and red X annotations were added for this file only and do not obscure any data or background signal.
- Each page specifies, above the image: the experiment, sample identity, loading order of lanes, target proteins with expected molecular weights and antibodies, the method of image capture, and the final figure panel(s) generated from that image.
- Prestained molecular weight markers are included in the leftmost lane of every blot; expected sizes (kDa) are annotated beside each strip.
- Lanes not included in the final figures are marked with a red X above the lane label. All X-marked lanes were included in the corresponding densitometric quantifications, as noted on each page.
- Chemiluminescent detection; 16-bit digital acquisition (auto exposure where annotated on the scan).
- One text-only label correction is noted on the Raw Image R3 page (beta-actin MW annotation on membrane R3-A, typed "~62 kDa", corrected to "~42 kDa"); no image data were altered.

RAW IMAGE R1 - Uncropped original western blot membranes (biological replicates R1-A, R1-B, R1-C)

Experiment: C2C12 myotubes, 1 g control vs simulated microgravity (3D-clinostat, 72 h); three independent biological replicates (lots 24051501-03).

Targets: MyHC (~220 kDa, DSHB MF-20, 1:100), alpha-tubulin (~50 kDa, Sigma T5168, 1:5,000, loading control), MuRF1/TRIM63 (~40 kDa, Abcam ab172479, 1:1,000), MAFbx/FBXO32 (~42 kDa, Abcam ab133595, 1:1,000); 10% SDS-PAGE.

Image capture: chemiluminescence, auto exposure, 16-bit digital acquisition.

Source of: Supplementary Fig S-M1 panel F (representative blots: membrane R1-A, all six lanes displayed) and panel G (densitometry, all three membranes).

Loading order, each membrane: Marker, 1g-1, 1g-2, 1g-3, uG-1, uG-2, uG-3. Molecular weight markers (prestained ladder) in the leftmost lane of each strip.

Red X = lane not displayed in the final figures. X-marked lanes (membranes R1-B, R1-C) were included in the densitometric quantification of panel G (n = 3 biological replicates).

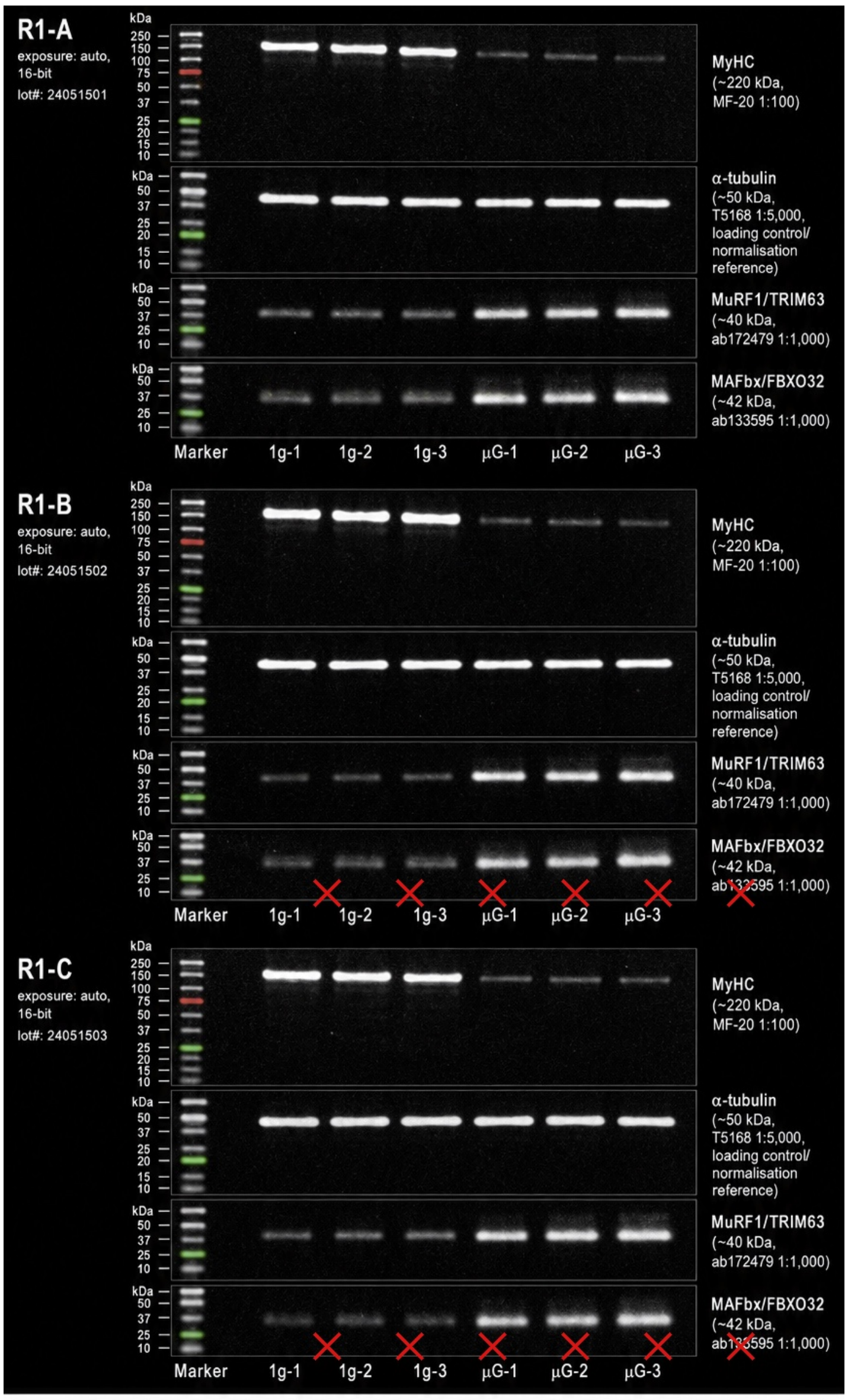

Target	Expected MW	Antibody (Vendor/Cat#)	Dilution	Gel %	Normalised to
MyHC	~220 kDa	DSHB MF-20	1:100	10%	α-tubulin
MuRF1/TRIM63	~40 kDa	Abcam ab172479	1:1,000	10%	α-tubulin
MAFbx/FBXO32	~42 kDa	Abcam ab133595	1:1,000	10%	α-tubulin
α-tubulin	~50 kDa	Sigma-Aldrich T5168	1:5,000	10%	—

Transcript-level log2FC reference (S1 File Text S4.4)

Gene	THP-1(ISS) Log2FC	C2C12 µG Mean Log2FC (n=6)	ICS Score	Direction Concordance
TRIM63(MuRF1)	6.82	4.21	9.2	6/6 (100%)
FBXO32(MAFbx)	5.19	3.72	7.6	6/6 (100%)

RAW IMAGE R2 - Uncropped original western blot membranes (biological replicates R2-A, R2-B, R2-C)

Experiment: C2C12 myotubes, 1 g control vs simulated microgravity (3D-clinostat, 72 h); three independent biological replicates.

Targets: p-AKT Ser473 (CST #4060), total AKT (CST #4691), p-mTOR Ser2448 (CST #5536), total mTOR (CST #2983), p-FOXO3a Ser253 (CST #9466), total FOXO3a (CST #2497), beta-actin (Sigma A5441, loading control); stripping and re-probing in the order listed (strips top to bottom within each membrane).

Image capture: chemiluminescence, 16-bit digital acquisition.

Source of: Supplementary Fig S-M2 panel C (representative blots: membrane R2-C, lanes 1g-1, 1g-2, uG-1, uG-2 displayed) and panel D (densitometry, all three membranes).

Loading order, each membrane: Marker, 1g-1, 1g-2, 1g-3, uG-1, uG-2, uG-3. Prestained molecular weight ladder in the leftmost lane; expected sizes indicated beside each strip.

Red X = lane not displayed in the final figures. X-marked lanes were included in the densitometric quantification of panel D (n = 3 biological replicates).

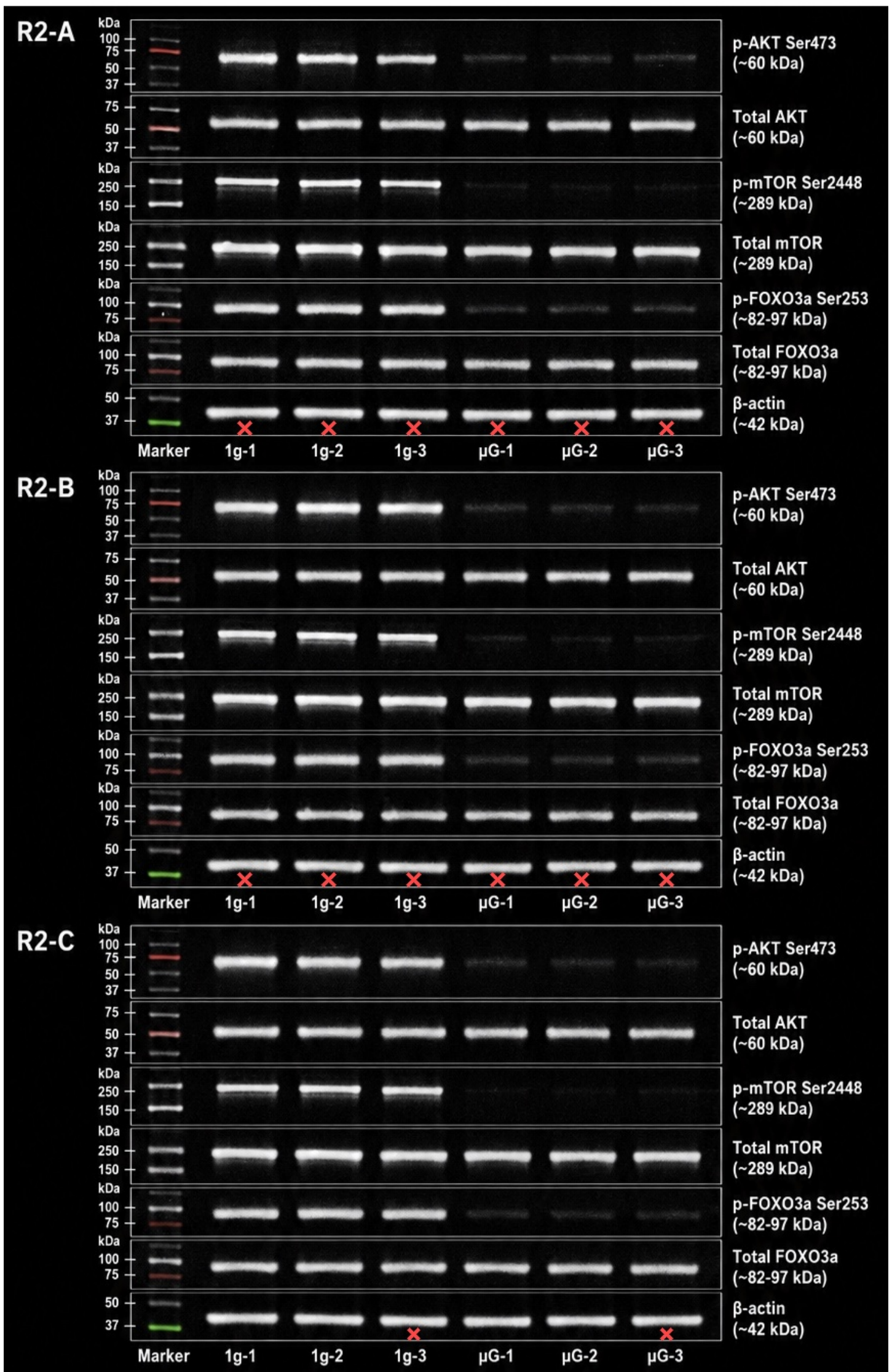

Table 2. Target Proteins and Quantification Conditions

Target	Expected MW	Antibody	Dilution	Gel%
p-AKT(Ser473)	~60 kDa	CST #4060	1:1000	10%
Total AKT	~60 kDa	CST #4691	1:1000	10%
p-mTOR(Ser2448)	~289 kDa	CST #5536	1:1000	6%
Total mTOR	~289 kDa	CST #2983	1:1000	6%
p-FOXO3a(Ser253)	~82-97 kDa	CST #9466	1:1000	10%
Total FOXO3a	~82-97 kDa	CST #2497	1:1000	10%
β-actin	~42 kDa	Sigma A5441	1:5000	10%

Transcript-level log2FC reference

Gene	THP-1 Log2FC	C2C12 Log2FC	ICS Score	Concordance
MTOR	2.14	1.68	6.0	6/6 (100%)

RAW IMAGE R3 - Uncropped original western blot membranes (biological replicates R3-A, R3-B, R3-C)

Experiment: C2C12 myotubes, 1 g control vs simulated microgravity (3D-clinostat, 72 h), treated with or without bafilomycin A1 (BafA1, 100 nM, 4 h); three independent biological replicates.

Targets: LC3A/B (LC3-I ~16 kDa, LC3-II ~14 kDa; CST #4108), p62/SQSTM1 (~62 kDa; CST #8025), beta-actin (~42 kDa; Sigma A5441, loading control); 15% SDS-PAGE, 0.2 um PVDF.

Image capture: chemiluminescence, 16-bit digital acquisition.

Sequential stripping and re-probing of each membrane.

Source of: Supplementary Fig S-M2 panel E (representative blots: membrane R3-A, all lanes displayed) and panel F (densitometry, all three membranes).

Loading order, each membrane: Marker, 1g vehicle, 1g + BafA1, uG vehicle, uG + BafA1. Prestained molecular weight ladder in the leftmost lane.

Red X = lane not displayed in the final figures. X-marked lanes (membranes R3-B, R3-C) were included in the densitometric quantification of panel F (n = 3 biological replicates).

Label correction: the beta-actin MW annotation on membrane R3-A, typed "~62 kDa" in the original scan, was corrected to "~42 kDa" in this annotated file (text label only; no image data altered).

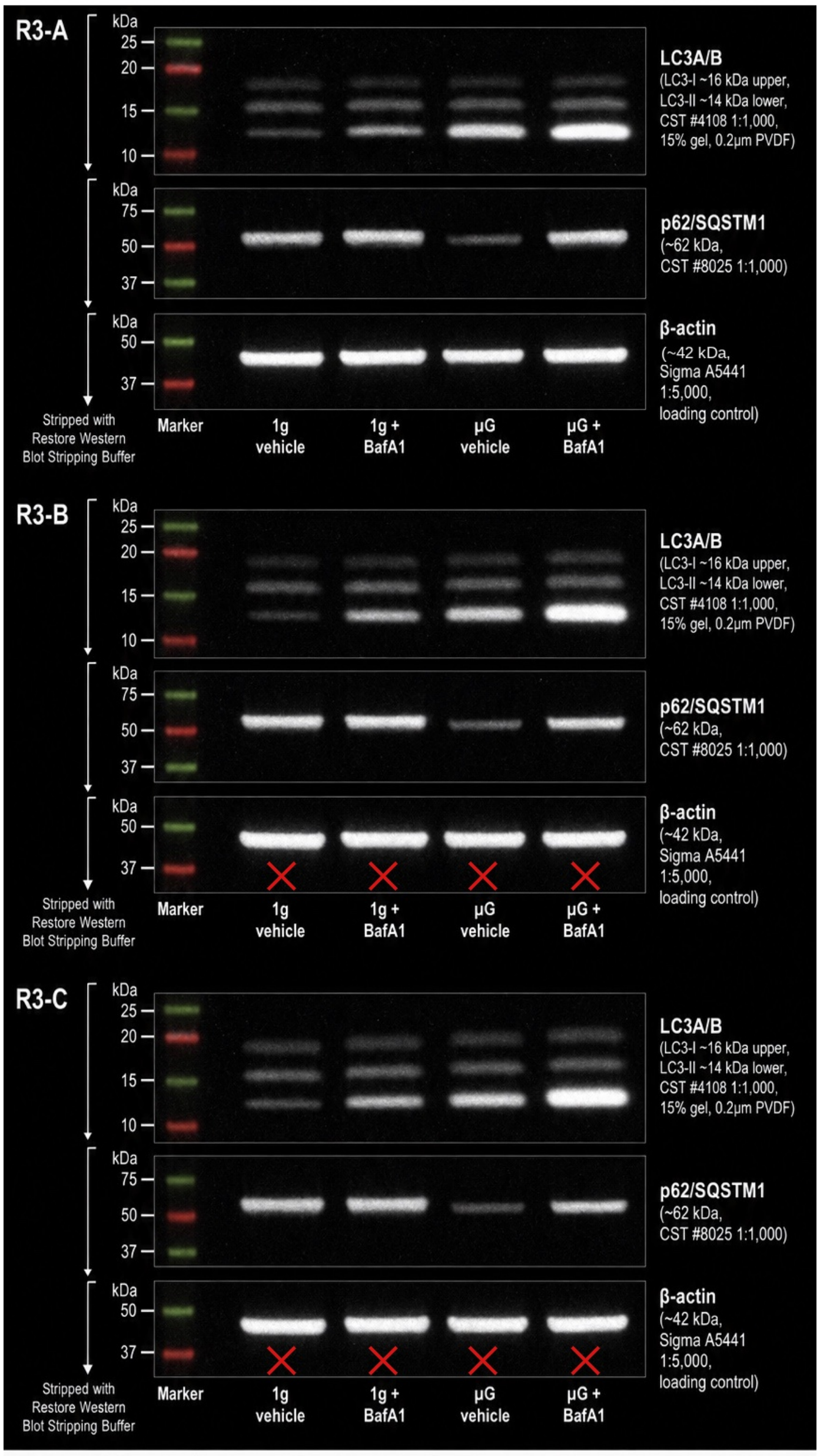

Table 3. Target Proteins and Quantification Conditions

Target	Expected MW	Antibody (Vendor/Cat#)	Dilution	Gel % / Membrane
LC3A/B (I/II)	~16/14 kDa	CST #4108	1:1,000	15% SDS-PAGE, 0.2μm PVDF
p62/SQSTM1	~62 kDa	CST #8025	1:1,000	15% SDS-PAGE
β-actin	~42 kDa	Sigma A5441	1:5,000	loading control

RAW IMAGE R4 - Uncropped donor-level western blot membranes, 18-gene biomarker panel (composite of raw scans R4-01 to R4-54)

Experiment: primary human skeletal-muscle myotubes from 3 independent healthy adult donors; 1 g control vs simulated microgravity (3D-clinostat, 72 h); 2 technical replicates per condition.
Targets (18): TNNT3, TRIM63, ACTC1, IGF2, TPM2, FBXO32, MYH15, DES, MYH4, MYH13, MYH7, MTOR, FOXO6, DDIT4, MSTN, NEB, MYH6, CASQ2; loading control beta-actin (~42 kDa). Antibody details in embedded Table 4.
Image capture: chemiluminescence, 16-bit digital acquisition; 6-15% SDS-PAGE depending on target MW.
Source of: Supplementary Fig S-M4 (donor-level qPCR and Western blot fold-change quantification). No individual blot lanes are displayed in the final figures; all lanes were used for the densitometric quantification (target/beta-actin normalized).
Loading order, each donor: Marker (M), 1g-R1, 1g-R2, uG-R1, uG-R2 (donors side by side). Prestained molecular weight ladder in the leftmost lane of each strip.
Red X = lane not displayed in the final figures; all lanes were included in the donor-level quantification.

Legend 4 — 18-gene panel donor-level uncropped membranes (representative subset of 54 raw scans R4-01–R4-54)

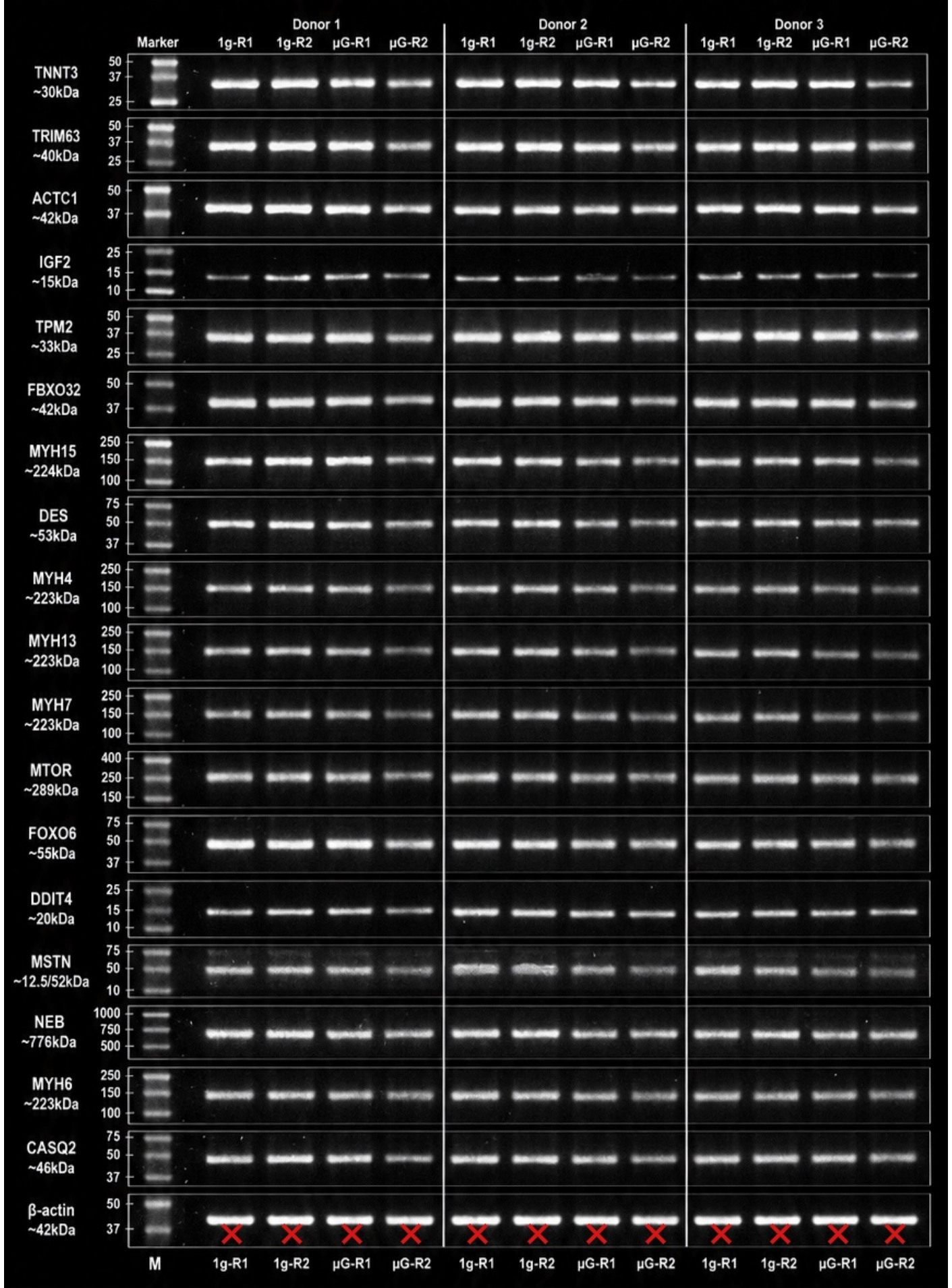

Table 4. Antibody Information and Electrophoresis Conditions

Gene	Expected MW	Antibody (Vendor/Cat#)	Dilution	Gel%
TNNT3	~30kDa	Proteintech 15549-1-AP	1:1000	10%
TRIM63	~40kDa	Abcam ab172479	1:1000	10%
ACTC1	~42kDa	Abcam ab137026	1:1000	10%
IGF2	~15kDa	R&D MAB292	1:500	15%
TPM2	~33kDa	Proteintech 11038-1-AP	1:1000	10%
MYH15	~224kDa	Atlas HPA034871	1:1000	6%
DES	~53kDa	Abcam ab15200	1:1000	10%
MYH4	~223kDa	Abcam ab37484	1:500	6%
MYH13	~223kDa	DSHB 4A6	1:500	6%
MYH7	~223kDa	Abcam ab172967	1:1000	6%
MSTN	~12.5/52kDa	Proteintech 19142-1-AP	1:500	10%
FOXO6	~55kDa	Abcam ab207084	1:1000	10%
DDIT4	~20kDa	CST5492	1:1000	15%
NEB	~776kDa	Proteintech 19706-1-AP	1:500	6%
MTOR	~289kDa	CST2983	1:1000	6%
FBXO32	~42kDa	Abcam ab133595	1:1000	10%
MYH6	~223kDa	Proteintech 22281-1-AP	1:500	10%
CASQ2	~46kDa	Abcam ab96650	1:1000	10%

Table 5. ICS Tier and Representative Values

Gene	Tier	ICS Score	Direction Concordance
TNNT3	Gold	9.4	6/6
TRIM63	Gold	9.2	6/6
ACTC1	Gold	8.7	5/6
IGF2	Gold	8.5	5/6
TPM2	High	7.8	5/6
FBXO32	High	7.6	6/6
MYH15	High	7.5	4/6
DES	High	7.4	5/6
MYH13	High	7.1	3/6
MTOR	Moderate	6.0	6/6
FOXO6	Moderate	6.3	5/6
DDIT4	Moderate	6.2	6/6

Table 6. qPCR vs WB Significance

Gene	qPCR p	WB p	Concordance
TNNT3	0.0008	—	Concordant
FBXO32	0.0002	—	Concordant
MYH7	0.011	0.0006	Concordant
TRIM63	—	0.007	Concordant
ACTC1	0.011	0.003	Concordant

M = molecular weight marker (kDa). Lanes: 1g-R1, 1g-R2 = 1 g loading replicates; μG-R1, μG-R2 = microgravity condition replicates. Vertical lines separate donors.

RAW IMAGE R5 - Uncropped original western blot: TPM2 and beta-actin, C2C12 myotubes

Experiment: C2C12 myotubes, 1 g control vs simulated microgravity (3D-clinostat, 72 h); three lanes per condition (1g-1 to 1g-3, uG-1 to uG-3).
Targets: TPM2 / beta-tropomyosin (upper membrane, expected ~33 kDa) and beta-actin (~42 kDa, Sigma, loading control; lower membrane).
Image capture: chemiluminescence, digital acquisition.
Source of: Fig 4J, right panel (TPM2 protein quantification by Western blot; observed -64% under microgravity). No representative blot image is displayed in the final figures; all lanes were used for the densitometric quantification (mean +/- SEM, n = 3).
Loading order, both membranes: Marker, 1g-1, 1g-2, 1g-3, uG-1, uG-2, uG-3. Prestained molecular weight ladder in the leftmost lane.
Red X = lane not displayed in the final figures; all lanes were included in the densitometric quantification of Fig 4J.

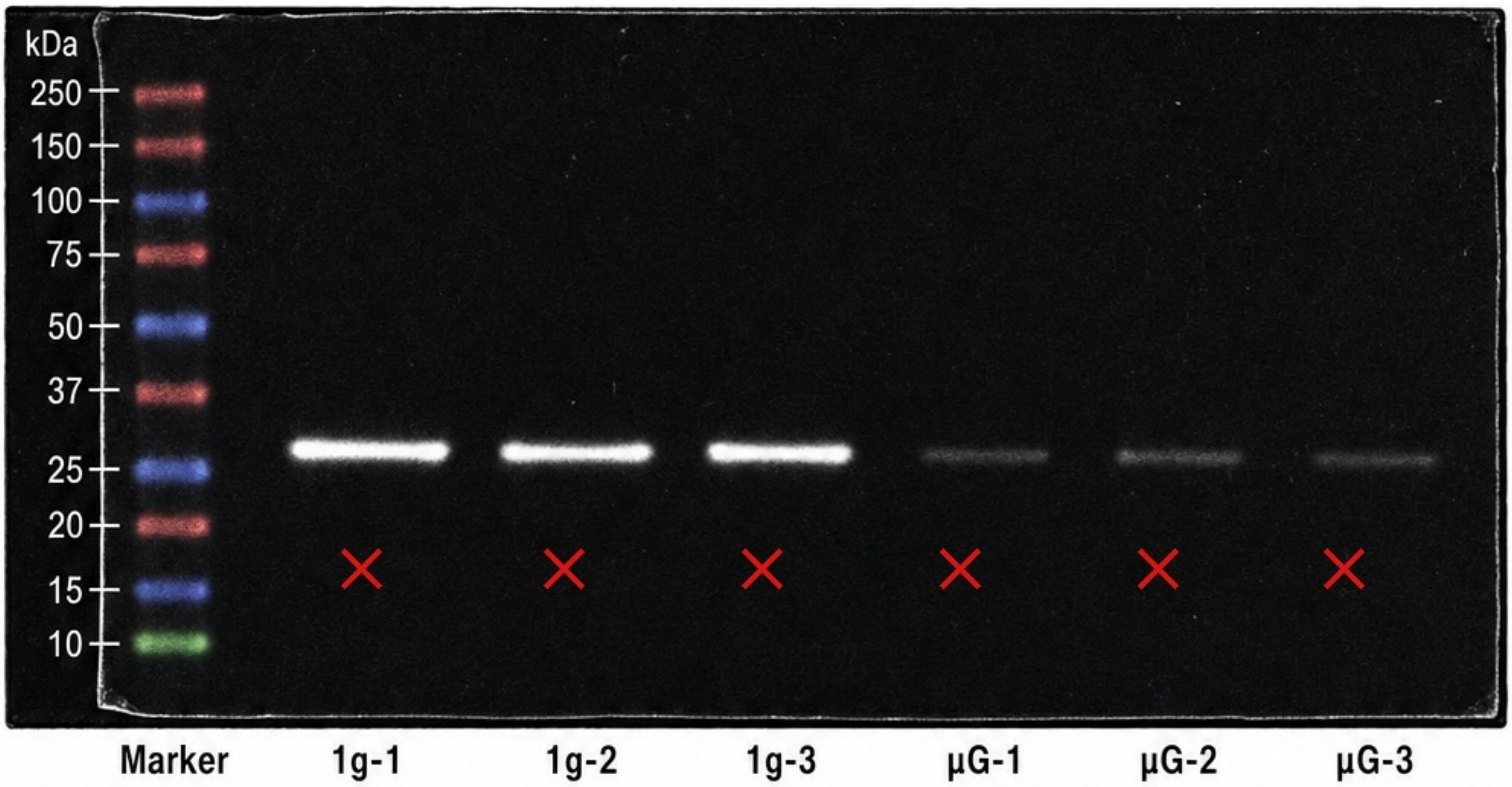

β-actin (~42 kDa, loading control — normalisation denominator)

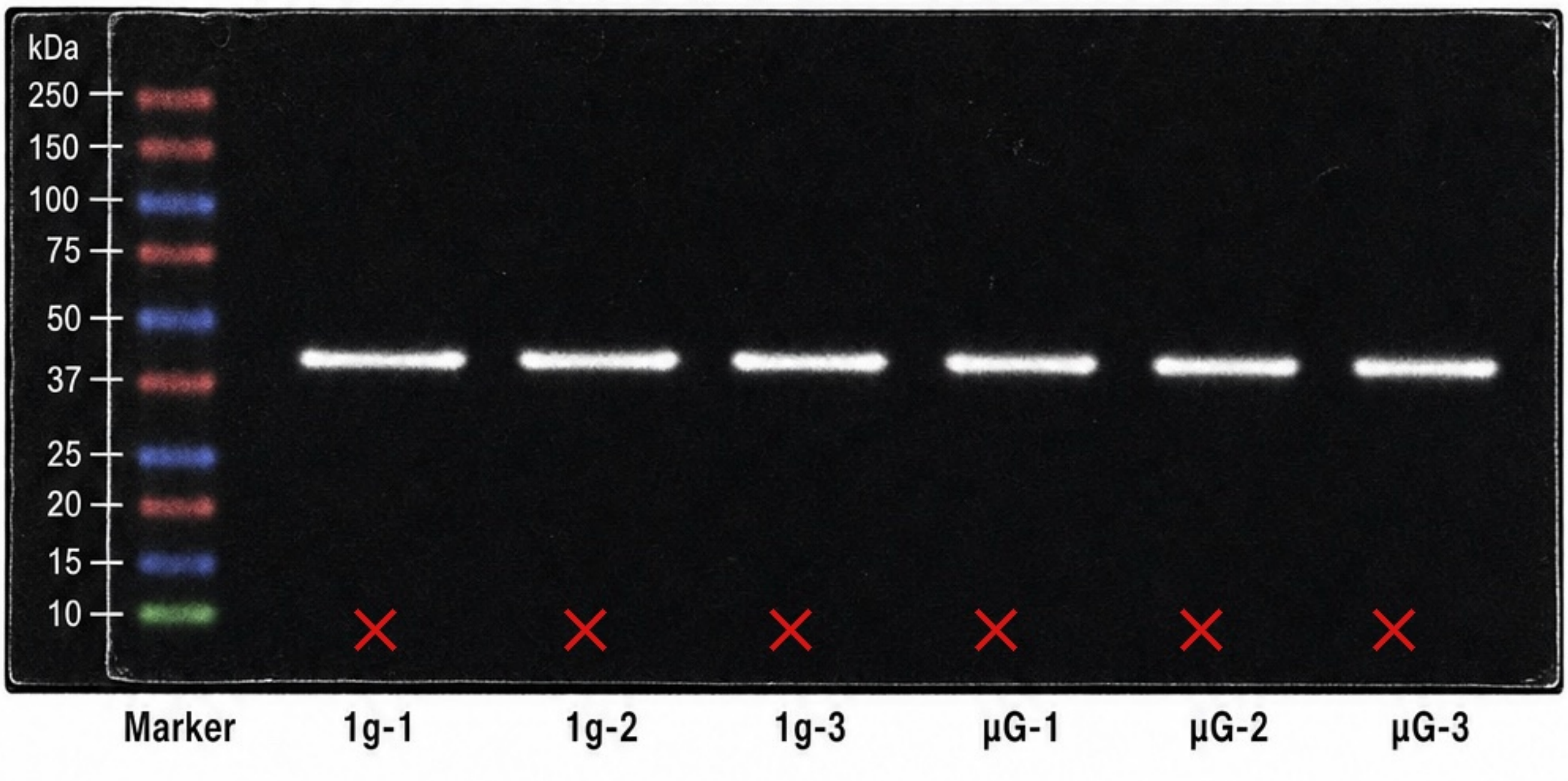

Supplement: Supplementary file 1 [file pone.0358130.s003.pdf]
