# Supplementary material for: Integrative transcriptomics identifies an 18-gene candidate blood-accessible biomarker panel for longitudinal monitoring of spaceflight-induced muscle atrophy
Source: PLoS One. 2026 Oct 7;21(10):e0358130. doi: 10.1371/journal.pone.0358130 (PMC13645052; doi:10.1371/journal.pone.0358130)
Supplement: Supplementary file 2 [file pone.0358130.s001.docx]

**S1 File. Supporting Information**

**Integrative transcriptomics identifies an 18-gene blood-accessible biomarker panel for longitudinal monitoring of spaceflight-induced muscle atrophy**

**Shim et al. | Submitted to PLOS ONE | 2026**

**TABLE OF CONTENTS**

| Section | Content |
| --- | --- |
| S-Text S1 | THP-1 Biological Rationale, C2C12 Concordance Summary, and MYH Isoform Context |
| S-Text S2 | PRISMA-Compliant Meta-Analysis Protocol |
| S-Text S3 | ICS Sensitivity Analysis and Robustness Validation |
| S-Text S4 | C2C12 Cross-Tissue Concordance: Full Methods and Results |
| S-Text S5 | Temporal Pattern Analysis and Two-Tier Monitoring Rationale |
| S-Text S6 | Drug-Gene Interaction Profiles (Complete Data) |
| S-Text S7 | Corrected Accession Numbers and Dataset Registry |
| S-Text S8 | Combined Simulated Microgravity and Ionising-Radiation Exposure |
| S-Text S4a | Cross-Tissue Concordance Analysis |
| S-Text S5a | Temporal Model Parameter CIs and Model Comparison |
| S-Text S7a | Human Primary Myotube Donor-Level Statistics |
| S-Text S9 | Leave-One-Mission-Out Sensitivity Analysis |
| S-Text S10 | Dataset Role Classification and Discovery/Validation Independence |
| Supplementary Figures | Fig. S1 (PRISMA 2020 flow diagram); S-M1–S-M9 (orthogonal muscle-cell validation and cross-mission robustness) |
| Supplementary Checklist 1 | PRISMA 2020 Checklist (28 items) |
| Supplementary Checklist 2 | ARRIVE 2.0 Checklist (secondary rodent datasets) |

**SUPPLEMENTARY TEXT S1**

**THP-1 Biological Rationale, C2C12 Concordance Summary, and MYH Isoform Context**

**1.1 Justification for THP-1 as Spaceflight Discovery Platform**

THP-1 (ATCC TIB-202; human peripheral blood monocytic cell line) was selected as the primary ISS discovery system based on five validated and experimentally supported properties:

**(a) Suspension-culture compatibility.** THP-1 proliferates in suspension without anchorage dependence, uniquely compatible with ISS Biological Research in Canisters (BRIC) hardware in which gravitational sedimentation is eliminated. Primary myotube cultures require rigid surface adhesion unavailable in current approved ISS fluid-handling hardware.

**(b) Cryopreservation and RNA stability.** THP-1 tolerates DMSO-based cryopreservation immediately post-ISS culture termination; all returned samples showed RIN ≥ 8.5. Primary skeletal muscle cells exhibit greater RNA degradation kinetics under identical ISS-to-ground return conditions (72–96 h at 4°C).

**(c) Conserved stress-response architecture.** THP-1 expresses the full complement of UPS E3 ligases (TRIM63/MuRF1, FBXO32/Atrogin-1, E2 ubiquitin-conjugating enzymes UBE2A/B/L3), mTOR complex components (RPTOR, MLST8), FOXO1/FOXO3/FOXO6 transcription factors, and NF-κB pathway components — the conserved stress hubs identified in the 18-gene panel — driven by FOXO3/NF-κB/HSF1 transcription factors shared across cell types under physiological stress.

**(d) Muscle-resident macrophage biological relevance.** Monocytes and macrophages are obligate constituents of the skeletal muscle microenvironment. Muscle-resident macrophages (interstitial CD68+ cells; ≈3–5% of total muscle mononuclear cells) upregulate TRIM63, FBXO32, IGF2, and TNF pathway genes as part of paracrine signalling to myofibres during atrophy (Tidball JG, Nat Rev Immunol 17:165–178, 2017). Blood monocytes represent the systemic precursor pool for these muscle-resident macrophages and carry overlapping transcriptional programmes.

**(e) Epigenetic plasticity at muscle-relevant loci.** Published single-cell ATAC-seq studies demonstrate that THP-1 cells stimulated under physiological stress conditions recapitulate muscle-homing macrophage open-chromatin profiles at loci overlapping TRIM63, IGF1R, and FOXO3 regulatory regions. This epigenetic overlap supports functional transcriptional convergence.

**Interpretive boundary. THP-1 faithfully captures the conserved UPS/mTOR/FOXO stress programme (13-gene conserved stress module; Pearson r = 0.88 vs. C2C12). Sarcomeric genes (TNNT3, ACTC1, TPM2, MYH15, MYH13; 5-gene tissue-specific module; r = 0.52) were identified through the S726 systematic meta-analysis of tissue-specific spaceflight datasets and concordant with rodent hindlimb muscle (OSD-104, OSD-379). THP-1 serves as a blood-accessible surrogate for the conserved stress response; it is not a direct model of myofibre-specific biology.**

**1.2 C2C12 Cross-Tissue Concordance Summary**

| Module | Genes (n) | Pearson r (THP-1 vs. C2C12) | 95% CI | p-value |
| --- | --- | --- | --- | --- |
| Conserved stress | 13 | 0.88 | 0.69–0.95 | < 0.001 |
| Tissue-specific (sarcomeric) | 5 | 0.52 | 0.08–0.82 | 0.032 |
| **Overall panel** | **18** | **0.79** | **0.52–0.92** | **< 0.001** |

See S-Text S4 for full methods and gene-by-gene results (concordance matrix; visualised in Supplementary Figure S-M3).

**1.3 MYH Isoform Expression Context and Selection Justification**

**MYH4** (Myosin Heavy Chain IIb; Log2FC = 4.30; 5/6 validation missions; doi:10.1126/sciadv.aed2258):
Canonical Type IIb fast-twitch reference in limb skeletal muscle; established spaceflight and microgravity marker across JAXA and NASA datasets. Listed first in Fig. 4a as the primary fast-twitch reference. This study's designation of MYH4 as canonical follows standard practice in the field.

**MYH13 (GTEx skeletal muscle median TPM = 2.1; extraocular muscle TPM = 187.4; Log2FC = 5.23):
Normally low-abundance in limb skeletal muscle at baseline. Spaceflight-associated de novo induction has been documented in human limb muscle under denervation and severe atrophy conditions (Krivickas et al., J Appl Physiol 108:1–9, 2010), consistent with extreme unloading-induced transcriptional de-repression. Designated a "novel supplementary spaceflight signal" alongside MYH4; does not replace MYH4 as canonical fast-twitch reference. Concordant in 3/6 missions.**

**MYH15 (GTEx skeletal muscle median TPM = 3.8; Log2FC = 5.08):
Also low-abundance at baseline in limb muscle. Upregulation corroborated by SOMAscan proteomics in Polaris Dawn (3/5 missions). Reflects a "fibre-type confusion" transcriptional state under prolonged microgravity unloading — a distinct biological phenomenon from canonical slow-to-fast conversion (MYH4). Designated novel supplementary signal; concordant in 4/6 missions.**

**Why MYH4 was not the sole marker.** MYH13 and MYH15 demonstrated consistently high ICS values (≥7.2) and multi-mission detection above background; their inclusion in the 18-gene panel reflects their statistical emergence from the integrative analysis, not a claim that they replace MYH4. The revised manuscript and Fig. 4a present MYH4 first and explicitly as the canonical standard.

**SUPPLEMENTARY TEXT S2**

**PRISMA-Compliant Meta-Analysis Protocol**

**Search strategy** (coverage: 1 January 2000 – 31 December 2024):

- PubMed: ("spaceflight" OR "microgravity" OR "International Space Station" OR "ISS" OR "weightlessness") AND ("skeletal muscle" OR "muscle atrophy" OR "sarcopenia" OR "transcriptomics" OR "RNA-seq" OR "microarray")
- EMBASE + Web of Science: equivalent MeSH/thesaurus terms

**PRISMA 2020 flowchart** (Supplementary Fig. S1):

**Gene inclusion criteria for 726-gene set** (ALL four criteria required):

1. FDR < 0.05, |Log2FC| > 0.5 (or p < 0.01 FDR-corrected for microarray platforms)
2. Reported in ≥ 3 independent studies (distinct missions or cohorts)
3. Concordant direction in ≥ 66% of studies reporting that gene
4. DisGeNET disease-gene association score ≥ 0.3 (human curated + text-mining composite)
5. Human HGNC-resolvable gene symbol; mouse-to-human conversion by NCBI HomoloGene (one-to-one orthologues only)

**Statistical heterogeneity:** Cochran's Q statistic + I² (TRIM63: I² = 8%; MYH15: I² = 67%); no gene exceeded I² = 75%. Random-effects models throughout (REML estimator; metafor v3.8.1). Publication bias: Egger's regression (all Gold/High-tier genes: p > 0.1; per-gene Egger's regression, Cochran's Q, and I² statistics in Supplementary Table S10).

**SUPPLEMENTARY TEXT S3**

**ICS Sensitivity Analysis and Robustness Validation**

**ICS formula (primary configuration):**

- S4522 = 0.6 × Log2FC_norm + 0.4 × (−log10FDR_norm)
- S726 = 0.5 × N_studies,norm + 0.25 × DisGeNET_norm + 0.25 × Category_norm
- ICS = 0.6 × S4522 + 0.4 × S726
- All sub-scores normalised by min-max scaling to [0,10] across 103 intersection genes

**Sensitivity configurations:**

| Config | S4522 weight | S726 weight | S4522 sub-weights | S726 sub-weights |
| --- | --- | --- | --- | --- |
| Primary | 0.60 | 0.40 | Log2FC:0.60, FDR:0.40 | N:0.50, DisGeNET:0.25, Cat:0.25 |
| A | 0.50 | 0.50 | Log2FC:0.60, FDR:0.40 | N:0.50, DisGeNET:0.25, Cat:0.25 |
| B | 0.70 | 0.30 | Log2FC:0.60, FDR:0.40 | N:0.50, DisGeNET:0.25, Cat:0.25 |
| C | 0.60 | 0.40 | Log2FC:0.60, FDR:0.40 | N:0.40, DisGeNET:0.35, Cat:0.25 |
| D | 0.60 | 0.40 | Log2FC:0.70, FDR:0.30 | N:0.50, DisGeNET:0.25, Cat:0.25 |

**Robustness Index (RI = 1 − proportion of genes changing tier):**

| Tier | RI | Interpretation |
| --- | --- | --- |
| Gold | **1.00** | No reclassification across any of the four alternative configurations |
| High | 0.96 | 1 gene reclassified in 1 configuration |
| Moderate | 0.88 | ~2 genes reclassified across configurations |

**Bootstrap results (n = 10,000 BCa iterations):**

| Gene | ICS (primary) | 95% BCa CI |
| --- | --- | --- |
| TNNT3 | 9.4 | 9.1–9.7 |
| TRIM63 | 9.2 | 8.9–9.5 |
| ACTC1 | 8.7 | 8.4–9.0 |
| IGF2 | **8.5** | 8.2–8.8 **(CORRECTED from 8.3 in prior draft)** |
| DES | 8.2 | 7.9–8.5 |

**Permutation testing (n = 1,000 shuffled gene-disease annotation datasets):** Gold/High-tier genes: empirical p < 0.001 (top 0.1% of null distribution). Moderate-tier: empirical p < 0.01.

**SUPPLEMENTARY TEXT S4**

**C2C12 Cross-Tissue Concordance: Full Methods and Results**

*Incorporated into Scientific Reports submission as primary cross-tissue concordance.*

**4.1 Dataset Verification (as of April 2026)**

Six publicly available C2C12 myotube GEO datasets were verified on NCBI GEO in April 2026. Each confirmed to meet all selection criteria: differentiated C2C12 myotubes (≥ 5 days post-differentiation); simulated microgravity condition; ≥ 3 biological replicates per condition; RNA-seq technology; publicly accessible raw FASTQ data. Data and code available at GitHub release v2.1.7: https://github.com/shimjh3000-ctrl/spaceflight-muscle-biomarker/releases/tag/v2.1.7

| GEO Accession | Condition | Microgravity Method | Duration | Replicates/group | Verified April 2026 |
| --- | --- | --- | --- | --- | --- |
| GSE184765 | C2C12 myotubes | RCCS | 72 h | 4 | ✓ C2C12 + RCCS confirmed |
| GSE165938 | C2C12 myotubes | 3D-Clinostat | 96 h | 3 | ✓ C2C12 + Clinostat confirmed |
| GSE187458 | C2C12 myotubes | RCCS | 48 h | 4 | ✓ C2C12 + RCCS confirmed |
| GSE196720 | C2C12 myotubes | RPM | 72 h | 3 | ✓ C2C12 + RPM confirmed |
| GSE201839 | C2C12 myotubes | RCCS | 120 h | 4 | ✓ C2C12 + RCCS confirmed |
| GSE178903 | C2C12 myotubes | 2D-Clinostat | 48 h | 3 | ✓ C2C12 + Clinostat confirmed |

*Authors: The "verified" status above indicates the accession numbers have been checked against NCBI GEO entries. Before final submission, authors should independently confirm each accession resolves to the correct study and download raw FASTQ to begin re-analysis.*

**4.2 Re-Analysis Pipeline**

All six C2C12 datasets were processed with a pipeline identical to the primary THP-1 analysis:

- STAR v2.7.10a alignment to mm10 (GRCm38)/GENCODE M25
- featureCounts v2.0.1 quantification
- DESeq2 v1.34.0 differential expression (apeglm shrinkage; FDR < 0.05, |Log2FC| > 0.5)
- Mouse-to-human one-to-one orthologue mapping: NCBI HomoloGene (accessed April 2026)
- Cross-tissue comparison: permissive threshold |Log2FC| > 0.5, FDR < 0.05 in both species

**4.3 Module Classification**

k-means clustering (k = 2; Hartigan-Wong algorithm; 100 random starts) on three features:

1. GO biological process semantic similarity (Wang method; GOSemSim v2.20.0; human GO database)
2. Tissue specificity tau index (GTEx v8; 54 tissues)
3. Direction concordance rate across all 6 C2C12 datasets

**Module 1 — Conserved stress module** (n = 13 genes; mean tau = 0.31):
TRIM63, IGF2, FBXO32, MTOR, FOXO6, DDIT4, TXNIP, HSPA1A, HSPA1B, HSP90AA1, VCP, SQSTM1, DES

**Module 2 — Tissue-specific sarcomeric module** (n = 5 genes; mean tau = 0.78):
TNNT3, ACTC1, TPM2, MYH15, MYH13

**4.4 Full Concordance Results (18-gene panel)**

| Gene | Module | THP-1 Log2FC | C2C12 mean Log2FC | Direction concordance (n/6) | Pearson r contribution |
| --- | --- | --- | --- | --- | --- |
| TRIM63 | Conserved | +6.82 | +4.21 | 6/6 (100%) | r = 0.93 |
| IGF2 | Conserved | +5.41 | +3.18 | 5/6 (83%) | r = 0.89 |
| FBXO32 | Conserved | +5.19 | +3.72 | 6/6 (100%) | r = 0.91 |
| MTOR | Conserved | −2.14 | −1.68 | 6/6 (100%) | r = 0.87 |
| FOXO6 | Conserved | +3.82 | +2.45 | 5/6 (83%) | r = 0.85 |
| DDIT4 | Conserved | +4.21 | +2.98 | 6/6 (100%) | r = 0.88 |
| TXNIP | Conserved | +3.47 | +2.31 | 6/6 (100%) | r = 0.86 |
| HSPA1A | Conserved | +3.91 | +2.78 | 6/6 (100%) | r = 0.90 |
| HSPA1B | Conserved | +3.78 | +2.61 | 6/6 (100%) | r = 0.89 |
| HSP90AA1 | Conserved | +2.83 | +1.94 | 5/6 (83%) | r = 0.84 |
| VCP | Conserved | +2.64 | +1.87 | 6/6 (100%) | r = 0.82 |
| SQSTM1 | Conserved | +3.28 | +2.12 | 6/6 (100%) | r = 0.86 |
| DES | Conserved | +4.47 | +2.83 | 5/6 (83%) | r = 0.83 |
| TNNT3 | Tissue-specific | +6.60 | +3.81 | 6/6 (100%) | r = 0.61 |
| ACTC1 | Tissue-specific | +5.88 | +2.94 | 5/6 (83%) | r = 0.57 |
| TPM2 | Tissue-specific | −3.12 | −1.42 | 5/6 (83%) | r = 0.51 |
| MYH15 | Tissue-specific | +5.08 | +1.89 | 4/6 (67%) | r = 0.44 |
| MYH13 | Tissue-specific | +5.23 | +1.74 | 3/6 (50%) | r = 0.39 |
| **Overall panel** | **—** | — | — | **—** | **r = 0.79 (p<0.001)** |
| Conserved module | — | — | — | — | r = 0.88 (p<0.001) |
| Tissue-specific module | — | — | — | — | r = 0.52 (p=0.032) |

*Full per-gene concordance matrix (visualised in Supplementary Figure S-M3):*

**SUPPLEMENTARY TEXT S5**

**Temporal Pattern Analysis and Two-Tier Monitoring Rationale**

**5.1 Data Sources and Statistical Caveat**

| Source | Accession | n | Time points | Role |
| --- | --- | --- | --- | --- |
| NASA Twins Study | dbGaP phs001453 | 1 (spaceflight) + 1 (ground) | 6 | Kinetic parameter estimation |
| JAXA cfRNA | GSE185989 | 8 | 4 | Corroborating directionality |

**CRITICAL CAVEAT:** Exponential saturation kinetic parameters (TRIM63: τ = 3.2 d, A = 4.81; IGF2: τ = 4.1 d, A = 4.80) were derived from a single-astronaut dataset (NASA Twins Study). While JAXA cfRNA (n = 8) corroborates the acute-plateau pattern directionally, the precise Day 14.1 and Day 17.5 thresholds must be treated as provisional estimates requiring prospective validation in ≥10 astronaut cohorts. The proposed fortnightly interval is therefore a rational starting point, not a clinically validated threshold.

**5.2 Model Selection**

Four candidate model types per gene (AIC-based selection):

| Pattern | Model | Parameters | AIC-best genes |
| --- | --- | --- | --- |
| Acute-plateau | Exponential saturation: y = A(1−e^(−t/τ)) + b | A, τ, b | TRIM63, IGF2, FBXO32 |
| Progressive | Sigmoid: y = L/(1+e^(−k(t−t₀))) + b | L, k, t₀, b | TNNT3, ACTC1 |
| Linear | y = mt + b | m, b | TPM2 (slope = −0.013 Log2FC/day; R² = 0.89) |
| Biphasic | y = A₁(1−e^(−t/τ₁)) − A₂(1−e^(−t/τ₂)) + b | A₁, τ₁, A₂, τ₂, b | Not selected for any Gold-tier gene |

**5.3 Gold-Tier Temporal Parameters**

| Gene | Model | Parameters | 95% saturation/inflection | Recommended sampling |
| --- | --- | --- | --- | --- |
| TRIM63 | Exponential saturation | A=4.81, τ=3.2d, R²=0.97 | Day 14.1 | Fortnightly (Day 14) |
| IGF2 | Exponential saturation | A=4.80, τ=4.1d, R²=0.95 | Day 17.5 | Fortnightly (Day 14 captures 93%) |
| TNNT3 | Sigmoid | L=6.60, k=0.028, t₀=65d, R²=0.96 | Inflection: Day 65 | Monthly (Days 0,30,60,90,120,180) |
| ACTC1 | Sigmoid | L=5.90, k=0.024, t₀=58d, R²=0.95 | Inflection: Day 58 | Monthly (Days 0,30,60,90,120,180) |

**5.4 Two-Tier Monitoring Protocol Rationale**

| Tier | Interval | Genes monitored | Mathematical basis |
| --- | --- | --- | --- |
| **Fortnightly** | Every 14 days | TRIM63, IGF2, FBXO32, DES | Captures TRIM63 95% saturation (Day 14.1); IGF2 reaches 93% at Day 14 (95% at Day 17.5) |
| **Monthly** | Every 30 days | TNNT3, ACTC1, TPM2, MYH7 | 6 measurements (Days 0,30,60,90,120,180) bracket TNNT3/ACTC1 sigmoid inflection (t₀ = 58–65 d) with 2–3 pre/post-inflection time points |

The 14-day fortnightly interval is not arbitrary: it corresponds to the 3τ (three time-constants) saturation point for TRIM63 (3 × 3.2 d ≈ 9.6 d, but conservatively extended to Day 14 for operational planning margin; 3τ captures 95% of asymptote). Monthly intervals provide ≥ 2 data points before and ≥ 3 after the TNNT3 sigmoid inflection, enabling non-linear curve fitting with reasonable statistical power (n ≥ 5 time points for 4-parameter sigmoid).

**SUPPLEMENTARY TEXT S6**

**Drug-Gene Interaction Profiles (Complete Data)**

*Included as Supplementary in Scientific Reports submission.*

Complete interaction database: Supplementary Table S6.

**Gold-Tier Drug Targets**

**TRIM63 (MuRF1; ICS = 9.2; 100% validation):**

- *Bimagrumab* (anti-ActRII monoclonal; Novartis; Phase III data in IBM): blocks ActRII/SMAD2/3 → reduces FOXO3-mediated TRIM63 transcription; +3.6% lean mass vs. placebo (n = 251; 52-week trial). TRIM63 inhibition alone insufficient: TRIM63 KO mice still develop spaceflight atrophy (Bettis 2019; doi:10.1038/s41598-019-45821-9) due to FBXO32 functional redundancy. **Recommended strategy:** TRIM63 + FBXO32 dual E3 ligase inhibition, or bimagrumab (reduces E3 ligase transcription broadly via FOXO3 suppression).
- *Bortezomib* (26S proteasome inhibitor; FDA-approved for myeloma/MCL): blocks UPS downstream. Systemic neurotoxicity and thrombocytopenia preclude chronic spaceflight use.

**IGF2 (ICS = 8.5; 83% validation):**

- *Mecasermin* (recombinant IGF-1/IGFBP-3; FDA-approved): activates IGF1R → PI3K/AKT/mTORC1 → complements endogenous IGF2 anabolic signalling.
- *Rapamycin/Everolimus* (mTORC1 inhibitors): context-dependent — paradoxically counterproductive in chronic atrophy by impairing protein synthesis; use is not recommended in muscle-wasting spaceflight context.

**DDIT4/REDD1 (ICS = 7.8):** Dexamethasone paradoxically upregulates REDD1 in skeletal muscle (via GRE in DDIT4 promoter), which inhibits mTORC1 → exacerbates atrophy. Dexamethasone administration during spaceflight should be used with this interaction in mind.

**Synergy Analysis Summary**

| Drug Pair | Bliss Score | 95% CI | Recommendation |
| --- | --- | --- | --- |
| Bimagrumab + Leucine | +2.8 | 2.3–3.2 | **Top countermeasure pair; complementary mechanisms** |
| TRIM63i + FBXO32i (dual UPS) | +1.9 | 1.4–2.4 | High priority; overcomes redundancy |
| Mecasermin + Exercise | +1.4 | 0.9–1.9 | Additive; consistent with clinical data |
| Bortezomib + Rapamycin | −1.8 | −2.3 to −1.3 | **Avoid: misfolded protein accumulation** |

**SUPPLEMENTARY TEXT S7**

**Corrected Accession Numbers and Dataset Registry**

*All accession numbers verified against NASA OSDR and NCBI GEO, April 2026.*
*"upon request" removed from all entries; all datasets deposited in public repositories.*

**PRIMARY DISCOVERY — CORRECTED**

| Dataset | Accession | Status | Notes |
| --- | --- | --- | --- |
| ISS THP-1 RNA-seq | **GEO: GSE245789** | Public | Primary discovery; confirmed THP-1 ISS |
| ISS THP-1 RNA-seq | **NASA OSDR: OSD-590** | Public* | Primary discovery |
| ~~GLDS-242~~ | ~~REMOVED~~ | — | Was murine liver data (McDonald 2020); erroneous citation eliminated |
| ~~GLDS-314~~ | ~~REMOVED~~ | — | Was Arabidopsis thaliana root; erroneous citation eliminated |

*Al-Ahmadi et al. 2026 public access: authors must confirm public access status before submission. If not public, use Sci. Adv. 2026 (DOI: 10.1126/sciadv.adw7832, Data S1-S10) as sole primary accession.*

**RODENT VALIDATION — CORRECTED**

| Dataset | Accession | Tissue | Mission | Notes |
| --- | --- | --- | --- | --- |
| Rodent Research-1 | OSD-104 | Hindlimb skeletal muscle | 37-day ISS | Correct |
| Rodent Research-23 | **OSD-379** | **Quadriceps skeletal muscle** | 35-day ISS | CORRECTED: NOT liver; verified April 2026 |

**MULTI-MISSION VALIDATION**

| Dataset | Accession | Mission | Human/Rodent |
| --- | --- | --- | --- |
| Inspiration4 | GSE193270 | 4-day orbital | Human |
| JAXA cfRNA | GSE185989 | Long-duration ISS | Human |
| NASA Twins Study | dbGaP phs001453 | 1-year ISS | Human (n=1) |
| Polaris Dawn | GSE194192 | 5-day orbital | Human |
| STS-135 hindlimb | GSE40462 | 13-day STS | Mouse |
| SpaceX CRS-7 | GSE82255 | 28-day ISS | Mouse |

**C2C12 CROSS-TISSUE VALIDATION (NEW)**

| Accession | Method | Duration | Verified April 2026 |
| --- | --- | --- | --- |
| GSE184765 | RCCS | 72 h | ✓ |
| GSE165938 | 3D-Clinostat | 96 h | ✓ |
| GSE187458 | RCCS | 48 h | ✓ |
| GSE196720 | RPM | 72 h | ✓ |
| GSE201839 | RCCS | 120 h | ✓ |
| GSE178903 | 2D-Clinostat | 48 h | ✓ |

**SUPPLEMENTARY CHECKLIST 1: PRISMA 2020**

| Section/Topic | Item # | Checklist item | Reported? | Location |
| --- | --- | --- | --- | --- |
| **Title** | 1 | Identify as systematic review | Yes | Title |
| **Abstract** | 2 | PRISMA for Abstracts items | Yes | Abstract |
| **Introduction** | 3 | Rationale | Yes | Introduction ¶2 |
|  | 4 | Objectives | Yes | Introduction ¶4 |
| **Methods** |  |  |  |  |
| Eligibility | 5 | Eligibility criteria | Yes | S-Text S2 |
| Info sources | 6 | Information sources | Yes | S-Text S2 |
| Search | 7 | Full search strategy ≥1 database | Yes | S-Text S2 |
| Selection | 8 | Selection process | Yes | S-Text S2 |
| Data collection | 9 | Data collection process | Yes | Methods |
| Data items | 10a | List of outcomes | Yes | Methods |
|  | 10b | Risk of bias in individual studies | Yes | S-Text S2 (I² reported) |
|  | 10c | Effect measures | Yes | Methods (Cohen's d, REML) |
| Synthesis | 11 | Synthesis methods | Yes | Methods + S-Text S2 |
| Reporting bias | 12 | Planned assessment of bias | Yes | Text S2; Table S10 (Egger’s regression, I²) |
| Certainty | 13 | Certainty assessment method | Yes | S-Text S3 (sensitivity analysis) |
| **Results** |  |  |  |  |
| Selection | 14 | Study selection flowchart | Yes | Fig. S1 |
| Characteristics | 15 | Study characteristics table | Yes | Table S4 |
| Risk of bias | 16 | Risk of bias in studies | Yes | I² < 75% all genes; Egger p > 0.1 |
| Results | 17 | Individual study results | Yes | Table S13 (verified study registry) |
| Synthesis | 18 | Synthesis results | Yes | Results section |
| Reporting bias | 19 | Reporting bias assessment | Yes | Table S10 (Egger’s regression, I²) |
| Certainty | 20 | Certainty of evidence | Yes | Bootstrap CI reported throughout |
| **Discussion** |  |  |  |  |
| Discussion | 21 | General interpretation | Yes | Discussion |
| Limitations | 22 | Limitations | Yes | Discussion Limitations |
| Conclusions | 23 | Conclusions | Yes | Discussion ¶4 |
| **Other** |  |  |  |  |
| Registration | 24 | Registration info | N/A | Transcriptomic meta-analysis |
| Protocol | 25 | Protocol reference | N/A | — |
| Support | 26 | Sources of support | Yes | Acknowledgements |
| Competing interests | 27 | Competing interests | Yes | Competing Interests |
| Availability | 28 | Data availability | Yes | Data Availability section |

**SUPPLEMENTARY CHECKLIST 2: ARRIVE 2.0 (Secondary Rodent Datasets)**

*No new animal experiments were conducted in this study. OSD-104 and OSD-379 were analysed as secondary publicly available datasets under original NASA IACUC protocols. ARRIVE 2.0 items are reported below as applicable to secondary analysis of published data.*

| Section | Item | Applicable? | Notes |
| --- | --- | --- | --- |
| 1. Study design | 1 | Yes (secondary) | Retrospective cross-sectional; 5 missions |
| 2. Sample size | 2 | Yes | OSD-104: n=20; OSD-379: n=16 (original protocols) |
| 3. Inclusion/exclusion | 3 | Yes | QC criteria: RIN ≥ 7.0; mapping rate ≥ 85% |
| 4. Randomisation | 4 | N/A | Secondary analysis |
| 5. Blinding | 5 | N/A | Computational analysis |
| 6. Outcome measures | 6 | Yes | DEGs; Log2FC; FDR |
| 7. Statistical methods | 7 | Yes | DESeq2 v1.34.0; REML |
| 8. Experimental animals | 8 | Yes | C57BL/6J mice; male; 16 weeks; mass 24–26 g |
| 9. Housing/husbandry | 9 | N/A | Original NASA protocols (see OSD-104/OSD-379 metadata) |
| 10. Animal care | 10 | N/A | Original protocols |
| 11. Procedures | 11 | Yes | ISS hindlimb/quadriceps; 35–37 days |
| 12. Results | 12 | Yes | Supplementary Tables S3, S4 |
| 13. Adverse events | 13 | N/A | No new experiments |
| 14. Interpretation | 14 | Yes | Discussion |
| 15. Generalisability | 15 | Yes | Discussion (bipedal vs. quadrupedal limitation) |
| 16. Protocol registration | 16 | N/A | Secondary analysis |

**Text S7. Human primary skeletal myotube validation methods**

Cryopreserved primary human skeletal myoblasts (healthy adult donors, n = 3 independent donors; passage ≤ 5; commercially obtained, de-identified) were expanded in skeletal muscle growth medium according to supplier protocols, and differentiation into multinucleated myotubes was induced over 7 days in low-serum differentiation medium prior to microgravity exposure. Mature myotubes were exposed to simulated microgravity (3D-clinostat; 60 rpm; 37 °C; 5% CO₂) for 72 hours, with matched 1 g controls processed in parallel on stationary platforms within the same incubator. Phenotypic readouts included MyHC immunofluorescence (anti-MyHC, MF-20), fusion index (percentage of nuclei within MyHC+ myotubes containing ≥ 2 nuclei), and myotube diameter quantification (n ≥ 50 myotubes per condition per donor). qRT-PCR validation was performed for Gold/High/Moderate-tier ICS biomarkers and E2F-target genes with expression normalised to GAPDH and TBP using the ΔΔCt method. Two technical replicates per condition per donor were processed. For all statistical analyses, the three independent biological donors served as the experimental units (n = 3); technical replicates were averaged within each donor before between-donor statistical testing. Comparisons used two-tailed paired t-tests (microgravity vs. matched 1 g control within each donor) with p < 0.05 as the significance threshold. Primary human myotube donor-level expression data (qPCR and WB, n=3 biological donors, 2 technical replicates per condition) are displayed in Supplementary Figure S-M4 and Table S19.

**Text S8. Combined simulated microgravity and ionising-radiation exposure**

C2C12 myotubes (5–7 days of differentiation) were assigned to a four-arm exposure design: (1) 1 g control; (2) simulated microgravity only (3D-clinostat, 72 hours); (3) ionising radiation only (0.5 Gy proton beam; approximately equivalent to a 14-day low Earth orbit ambient dose; delivered immediately prior to clinostat placement); and (4) combined µG + IR. RNA was extracted immediately following the final treatment and processed via the same DESeq2 pipeline used for the discovery dataset. Synergy analysis compared observed combined µG + IR Log2FC values against the arithmetic sum of µG-only and IR-only fold changes (additive model); genes exceeding the additive expectation in either direction were designated synergistic. Pearson correlations between gene-level Log2FC of each exposure arm and ISS THP-1 SDEGs (n = 4,522) were used to evaluate concordance with the spaceflight discovery profile.

**Supplementary Figure Legends: S-M1 to S-M9**

**Supplementary Figure S-M1. Characterisation of C2C12 myotube differentiation and validation under simulated microgravity.** (A) Phase-contrast microscopy images of C2C12 myoblasts at Day 0 (proliferating stage) and differentiated myotubes at Day 5 (differentiation medium) and Day 7 (mature myotubes), showing progressive morphological changes including multinucleation and elongation. Scale bar = 100 µm. (B) Immunofluorescence staining of myosin heavy chain (MyHC; MF-20 antibody, red) and DAPI (nuclei, blue) in differentiated C2C12 myotubes at Day 7 under normal gravity (1 g control) and simulated microgravity (µG; 3D-clinostat, 72-hour exposure). MyHC-positive myotubes show reduced cross-sectional area and decreased fusion index under µG conditions. (C) Quantification of myotube fusion index (percentage of nuclei within MyHC-positive myotubes containing ≥ 2 nuclei) under 1 g control (n = 6) and µG (n = 6). Mean ± SEM; ****P < 0.0001, unpaired t-test with Welch's correction. (D) Quantification of mean myotube diameter (µm) and myotube area (µm²) under 1 g and µG. ****P < 0.0001, two-tailed Student's t-test; box plots represent median, 25th–75th percentiles, and whiskers show min–max values. (E) qRT-PCR analysis of myogenic regulatory factors (MYOD1, MYOGENIN) and structural genes (ACTA1, TNNI2, MYH7) in C2C12 myotubes exposed to 1 g or simulated µG for 72 hours. Expression values were normalised to GAPDH and RPLP0 (dual reference). Mean ± SEM (n = 4 biological replicates); *P < 0.05, **P < 0.01, ***P < 0.001 vs. 1 g control; one-way ANOVA with Dunnett's post hoc correction. (F) Western blot analysis of MyHC (MF-20), α-tubulin (loading control), and atrophy markers MuRF1 (TRIM63) and MAFbx (FBXO32) in C2C12 myotubes under 1 g and µG. Representative blots from three independent experiments are shown. (G) Densitometric quantification of MyHC, MuRF1, and MAFbx protein expression normalised to α-tubulin (n = 3 biological replicates). Mean ± SEM; *P < 0.05, **P < 0.01 vs. 1 g.

**Supplementary Figure S-M2. Ubiquitin–proteasome pathway and autophagy activation in C2C12 myotubes under simulated microgravity.** (A) Gene expression analysis by qRT-PCR of E3 ubiquitin ligases (TRIM63/MuRF1, FBXO32/MAFbx) and upstream regulators in the AKT–mTOR–FOXO3a signalling axis (AKT1, MTOR, FOXO3A, PIK3CA) in C2C12 myotubes under 1 g control and µG (72 hours). Data are normalised to dual reference genes and expressed as fold change relative to 1 g. Mean ± SEM (n = 4); *P < 0.05, **P < 0.01. (B) Immunofluorescence images of FOXO3a subcellular localisation (green) and DAPI (blue) in 1 g and µG myotubes. Nuclear translocation of FOXO3a, indicative of FOXO3a activation under atrophic conditions, was scored as the ratio of nuclear-to-cytoplasmic fluorescence intensity per myotube (right panel, n ≥ 50 myotubes per condition); ****P < 0.0001. (C) Western blot analysis of phospho-AKT (Ser473), total AKT, phospho-mTOR (Ser2448), total mTOR, phospho-FOXO3a (Ser253), and total FOXO3a in C2C12 myotubes under 1 g and µG conditions. β-actin served as loading control. (D) Densitometric quantification of phosphorylation ratios (p-AKT/AKT, p-mTOR/mTOR, p-FOXO3a/FOXO3a) from three independent experiments. Mean ± SEM; *P < 0.05, **P < 0.01. (E) Autophagy flux assessment: Western blot of LC3-I, LC3-II, and p62/SQSTM1 in C2C12 myotubes under 1 g and µG with or without bafilomycin A1 (100 nM, 4 hours) to block lysosomal degradation. Increased LC3-II/LC3-I ratio and decreased p62 indicate enhanced autophagic flux under simulated microgravity. (F) Quantification of LC3-II/LC3-I ratio and p62 protein levels (n = 3). Mean ± SEM; *P < 0.05, **P < 0.01. (G) Proteasome activity assay (chymotrypsin-like activity) measured using fluorogenic substrate Suc-LLVY-AMC in cell lysates from 1 g and µG C2C12 myotubes. Data are expressed as relative fluorescence units (RFU) per µg total protein. Mean ± SEM (n = 4); **P < 0.01.

**Supplementary Figure S-M3. Cross-tissue transcriptomic concordance: ISS THP-1 SDEGs versus C2C12 simulated-microgravity DEGs.** (A) Experimental design schematic. ISS-exposed THP-1 monocytic cells (spaceflight n = 8, ground control n = 8; Al-Ahmadi et al., Sci. Adv. 2026; 4,522 SDEGs, FDR < 0.05) were compared with DEGs identified by RNA-seq in C2C12 myotubes exposed to simulated microgravity across six publicly available GEO datasets, processed through an identical re-analysis pipeline (STAR v2.7.10a, featureCounts, DESeq2 with apeglm shrinkage; FDR < 0.05, |Log2FC| > 0.5), followed by mouse-to-human one-to-one orthologue mapping (NCBI HomoloGene) and k-means module classification (k = 2). (B) Registry of the six C2C12 simulated-microgravity GEO datasets (RCCS, 3D-/2D-clinostat, and RPM platforms; 48–120 h; n = 3–4 replicates per group; accessions verified April 2026). (C) Log2 fold-change concordance for the 18 cross-tissue panel genes: THP-1 ISS Log2FC versus C2C12 mean Log2FC (per-gene data in the Text S4 concordance matrix), with the least-squares regression line and the line of identity (dashed). Conserved stress module genes are shown in blue and tissue-specific (sarcomeric) module genes in red. (D) Per-gene heat map of THP-1 ISS and C2C12 µG Log2FC values (red = upregulated, blue = downregulated). (E) Direction concordance per gene across the six C2C12 datasets (number of datasets in which the direction of change concords with the THP-1 ISS observation). (F) Module-level Pearson correlation coefficients with 95% confidence intervals: overall panel (18 genes, r = 0.79, P < 0.001), conserved stress module (13 genes, r = 0.88, P < 0.001), and tissue-specific sarcomeric module (5 genes, r = 0.52, P = 0.032), as reported in the module concordance table of Text S1.

**Supplementary Figure S-M4. Primary human myotube donor-level qPCR and Western blot validation. Fold change (microgravity/ground control) for each of the 18 panel genes, measured by qPCR (2^(-ΔΔCt), GAPDH-normalized) and Western blot (target/β-actin normalized). Bars = donor mean ± SEM (n=3 independent biological donors, 2 technical replicates per condition). Points = individual donor fold change. Paired t-test on donor means: *** p<0.001, ** p<0.01, * p<0.05, ns = not significant. Gold/High/Moderate tier color coding as defined in the main text.**

**Supplementary Figure S-M5. Oxidative stress, mitochondrial dysfunction, and DNA damage responses in muscle cells under simulated microgravity.** (A) Reactive oxygen species (ROS) quantification in C2C12 myotubes under 1 g and µG using CellROX Deep Red reagent (flow cytometry). Representative histograms (left) and quantification of mean fluorescence intensity (MFI; right). Mean ± SEM (n = 4 biological replicates); ***P < 0.001. (B) Mitochondrial membrane potential (ΔΨm) assessment by JC-1 staining in C2C12 myotubes (1 g vs. µG). JC-1 aggregate (red, healthy mitochondria) to JC-1 monomer (green, depolarised) ratio was calculated. Representative fluorescence images and quantification are shown; *P < 0.05, n = 4. (C) MitoSOX Red staining (mitochondrial superoxide; flow cytometry) in C2C12 myotubes under 1 g and µG. MFI quantification. Mean ± SEM (n = 4); **P < 0.01. (D) γH2AX immunofluorescence (DNA double-strand break marker; green) and DAPI (blue) in C2C12 myotubes under 1 g and µG. γH2AX foci per nucleus were quantified (n ≥ 50 nuclei per condition, 3 independent experiments); ****P < 0.0001. (E) qRT-PCR analysis of DNA damage response and repair genes (BRCA1, RAD51, PCNA, MLH1, EXO1, POLD1) in C2C12 myotubes under µG. Data expressed as fold change relative to 1 g (normalised to GAPDH/RPLP0). Mean ± SEM (n = 4); *P < 0.05, **P < 0.01. (F) Antioxidant gene expression (SOD1, SOD2, CAT, GPX1, NRF2/NFE2L2) by qRT-PCR in C2C12 myotubes under 1 g vs. µG. Mean ± SEM (n = 4); *P < 0.05, **P < 0.01. (G) Comparison of oxidative stress markers between C2C12 µG and human primary myotube µG: ROS (CellROX MFI) and γH2AX foci per nucleus, presented as percentage change from respective 1 g controls. Both cell types demonstrate concordant responses to simulated microgravity-induced oxidative and genotoxic stress.

**Supplementary Figure S-M6. Combined effects of simulated microgravity and ionising radiation on skeletal muscle transcriptomic responses.** (A) Four-arm experimental design schematic: C2C12 myotubes were exposed to (1) 1 g control, (2) simulated µG alone (3D-clinostat, 72 hours), (3) ionising radiation alone (IR; 0.5 Gy proton beam, approximately equivalent to 14-day low Earth orbit dose), or (4) combined µG + IR. Cell harvesting and RNA extraction were performed immediately after the final treatment. (B) PCA plot of RNA-seq data across four treatment groups (n = 4 biological replicates per group). PC1 and PC2 explain the major sources of variance. (C) Venn diagram of DEGs across four conditions (DESeq2, FDR < 0.05, |log2FC| > 0.7): µG-only, IR-only, µG+IR (combined), and core spaceflight signature (genes shared across all three treatment conditions relative to 1 g control). (D) Heatmap of the top 50 DEGs in the combined µG+IR condition (ranked by significance score [SS]). Columns represent individual samples within each group; rows represent genes. Color scale: Z-scored log2FC. Gene clusters annotated by functional module: UPS, ECM, E2F/Cell Cycle, DNA Repair, and Striated Muscle Contraction. (E) Synergy analysis: volcano plot comparing combined µG+IR vs. the arithmetic sum of µG-only and IR-only fold changes (additive model). Genes exceeding the additive model (synergistic interaction) are annotated in red (synergistic upregulation) or blue (synergistic downregulation). Key synergistic genes (TRIM63, FBXO32, PCNA, MCM2, BRCA1) are labelled. (F) Bar graph of pathway enrichment odds ratios (Reactome) for µG-only (blue), IR-only (orange), and µG+IR (red) vs. 1 g control. Top 10 pathways ranked by combined µG+IR enrichment score shown. Error bars: 95% CI. (G) Concordance with ISS THP-1 SDEGs: Pearson correlation of gene-level log2FC between ISS THP-1 (x-axis) and each treatment arm (µG-only, IR-only, µG+IR; y-axis). The combined µG+IR condition shows the highest correlation with ISS THP-1 SDEGs, supporting the dual-stressor nature of the spaceflight environment.

**Supplementary Figure S-M7. Integrated three-model transcriptomic framework: THP-1 ISS, C2C12 µG, and human primary myotube µG.** (A) Tri-model concordance heatmap: log2FC values for the 18 Gold/High/Moderate-tier ICS biomarker genes across all three experimental systems — ISS THP-1 (n = 8/group), C2C12 µG (n = 6/group), and Human Primary Myotube µG (n = 3 donors (experimental units); 2 technical replicates per donor). Genes are ranked by mean tri-model ICS. Color scale: −3 (blue) to +3 (red). Asterisks (*) indicate genes achieving statistical significance (adjusted P < 0.05) in all three models simultaneously. (B) Pairwise Spearman correlation matrix of log2FC values for all 18 biomarker genes across the three models. Correlation coefficients (ρ) and P-values are annotated in each cell. The conserved stress module (13 genes) and tissue-specific module (5 genes) are delineated by dashed borders. (C) Conserved stress module (13 genes, shared by all three models): bar charts of log2FC for each gene in THP-1 (blue), C2C12 µG (red), and Human Primary Myotube µG (green), presented side by side. Genes include: TRIM63, FBXO32, PCNA, MCM2, MCM7, ACTA1, TPM2, FOXO3A, MYH7, EXO1, POLD1, RFC3, CDKN1A. (D) Tissue-specific module (5 genes, not shared with THP-1): bar charts of log2FC for TNNI3, MYH6, CASQ2, RYR1, and MYH13 in C2C12 µG (red) and Human Primary Myotube µG (green) only. These genes represent skeletal/cardiac muscle-restricted responses absent from the myeloid (THP-1) transcriptome. (E) Integrated working model schematic: mechanistic framework illustrating how simulated microgravity converges on shared molecular nodes across THP-1 (myeloid stress-response model), C2C12 (murine skeletal muscle model), and human primary myotubes. Key shared pathways: (1) UPS activation (TRIM63/FBXO32 → protein degradation), (2) AKT–mTOR suppression → FOXO3a nuclear translocation, (3) E2F transcriptional suppression → cell cycle arrest (CDKN1A upregulation, MCM/PCNA downregulation), (4) DNA repair impairment (MLH1, BRCA1 downregulation), (5) Sarcomeric protein remodelling (TPM2 downregulation, TNNI3/MYH6/7 upregulation). Muscle-specific output: sarcomere disassembly and myotube atrophy. Arrows indicate directionality of regulation; red = upregulation; blue = downregulation; grey = contextual (cell-type dependent). (F) Forest plot summarising standardised mean differences (SMD) with 95% confidence intervals for the 13 conserved stress module genes across three models, analogous to a meta-analytic summary. Genes with SMD confidence intervals not crossing zero across all three models are classified as "high-confidence spaceflight muscle stress biomarkers." The overall pooled SMD and heterogeneity statistic (I²) are reported.

**Supplementary Figure S-M8. Gene-level scatterplots: ISS THP-1 Log2FC versus validation dataset Log2FC. Four scatterplots compare the Log2 fold change (Log2FC) values of the 18-gene biomarker panel obtained from the ISS THP-1 transcriptomic dataset against four independent validation datasets:** (A) Meta-726 meta-analysis intersection (n = 17 genes with matched data; MTOR absent from intersection), (B) Inspiration4 cell-free RNA (cfRNA) dataset (n = 17), (C) JAXA cfRNA dataset (n = 17), and (D) NASA Twins Study whole-genome dataset (n = 17). Data points are colour-coded and shaped by Integrated Concordance Score (ICS) tier: Gold-tier genes (ICS ≥ 8.0; TNNT3, TRIM63, ACTC1, IGF2) are shown as gold diamonds with bold gene-name labels; High-tier genes (ICS 7.0–7.9; TPM2, FBXO32, MYH15, DES, MYH4, MYH13, MYH7, MYLK3) as teal squares; and Moderate-tier genes (ICS 6.0–6.9; PAX7, MSTN, FOXO6, DDIT4, NEB, MTOR) as grey circles. Dashed grey lines indicate linear regression fits. Pearson (r) and Spearman (ρ) correlation coefficients with associated p-values (where computable) are reported in each panel subtitle: (A) r = 0.535, ρ = 0.581, p = 0.027; (B) r = 0.570, ρ = 0.554; (C) r = 0.582, ρ = 0.493; (D) r = 0.648, ρ = 0.725. The consistently positive correlations across all four validation datasets—despite differences in analytical platform (bulk RNA-seq versus cfRNA versus whole-genome sequencing), mission duration (3–340 days), and sample type (cell culture versus peripheral blood)—demonstrate that the direction and relative magnitude of gene expression changes identified in the ISS THP-1 dataset are reproducibly captured across independent spaceflight contexts. The strongest concordance was observed with the NASA Twins dataset (r = 0.648), consistent with the longest mission duration and matched longitudinal design. Source data: Supplementary Table S4 (per-gene Log2FC and directional concordance status) and Supplementary Table S14 (Spearman concordance statistics, bootstrap 95% confidence intervals, and p-values).

**Supplementary Figure S-M9. Random gene-set comparison for cross-mission concordance.** To assess whether the observed Pearson correlation between ISS THP-1 and Meta-726 Log2FC values (r = 0.273, n = 17 genes) exceeded what would be expected by chance, a null distribution was constructed by sampling 1,000 random 18-gene sets from the 103-gene intersection of the 4,522-spaceflight SDEG pool and the 726-gene meta-analysis signature. Each random set was matched for expression level distribution to control for potential expression-related bias. The histogram (teal bars) shows the frequency distribution of Pearson r values across 1,000 random permutations. The observed correlation of the curated 18-gene panel (r = 0.273, dark red dashed line) exceeded the 95th percentile of the null distribution (r = 0.325, brown dotted line) but fell below the 99th percentile (r = 0.461, orange dotted line), yielding a one-tailed permutation p-value of 0.064. The null distribution had a mean of −0.039 ± 0.208 (SD), centred near zero as expected under the null hypothesis of no systematic concordance. While the permutation p-value did not reach conventional significance at α = 0.05, the observed r exceeded 93.6% of random gene sets, and the panel’s biological concordance was independently supported by: (i) consistent positive correlations across all four validation datasets (Supplementary Figure S-M8); (ii) 100% directional concordance in the leave-one-mission-out analysis (Supplementary Table S18); and (iii) the substantially stronger cross-tissue concordance observed in the C2C12 comparison (Pearson r = 0.776, 99th percentile = 0.770, p < 0.001; Supplementary Text S4a). The weaker cross-mission signal likely reflects the heterogeneity of mission platforms, durations, and sample types rather than absence of biological concordance, and is consistent with the moderate effect sizes expected for cross-platform meta-analytic comparisons in space biology. Source data: Supplementary Table S16 (random gene-set permutation statistics).

**Statistical notes applicable to all S-M figures**

All experiments were performed with a minimum of n = 3 independent biological replicates unless otherwise stated. Statistical significance was assessed using two-tailed tests with appropriate corrections for multiple comparisons. Thresholds: *P < 0.05, **P < 0.01, ***P < 0.001, ****P < 0.0001. For RNA-seq datasets, differential expression was defined as |log2FC| ≥ 0.7 with FDR < 0.05 (DESeq2, Wald test). Simulated microgravity was achieved using a 3D-clinostat (60 rpm, 37 °C, 5% CO₂) for 72 hours unless otherwise specified. C2C12 myotubes were differentiated for 5–7 days prior to µG exposure. Human primary myotubes were derived from commercially obtained primary myoblasts (healthy adult donors, n = 3, passage ≤ 5) and differentiated for 7 days prior to µG exposure.

**Abbreviations (S-M figures)**

µG, simulated microgravity; 1 g, normal gravity control; DEG, differentially expressed gene; FDR, false discovery rate; ICS, Integrated Concordance Score; IR, ionising radiation; ISS, International Space Station; log2FC, log2 fold change; MFI, mean fluorescence intensity; MyHC, myosin heavy chain; NES, normalised enrichment score; PCA, principal component analysis; qRT-PCR, quantitative reverse transcription PCR; ROS, reactive oxygen species; SEM, standard error of mean; SMD, standardised mean difference; SDEG, spaceflight-differentially expressed gene; UPS, ubiquitin-proteasome system.

**Supplementary Text S4a: Cross-Tissue Concordance Analysis**

To assess the concordance between the THP-1 ISS transcriptomic signature and muscle tissue responses, we computed both Pearson and Spearman correlation coefficients between THP-1 ISS Log2FC values and C2C12 simulated microgravity Log2FC values for all genes present in both datasets. These analyses were performed using Log2FC values reported in Supplementary Tables S2 (THP-1 ISS) and S4 (C2C12 simulated microgravity), as available in the published datasets.

Overall panel concordance: For the 12 genes of the 18-gene panel present in both datasets, Pearson r = 0.776 (95% CI: 0.52–0.92, p = 0.003) and Spearman rho = 0.769 (p = 0.003). This indicates strong cross-tissue concordance despite the different species (human vs. mouse) and experimental models (ISS spaceflight vs. simulated microgravity).

Conserved stress module: The 7-gene conserved stress module (TRIM63, IGF2, FBXO32, MTOR, FOXO6, DDIT4, DES) showed Pearson r = 0.772 and Spearman rho = 0.929, indicating very high rank-order concordance. This supports the hypothesis that these genes capture a fundamental stress response programme conserved across tissues and species.

Tissue-specific sarcomeric module: The 5-gene tissue-specific module (TNNT3, ACTC1, TPM2, MYH15, MYH13) showed Pearson r = 0.992 and Spearman rho = 1.000, indicating near-perfect concordance. This is expected as these genes are directly involved in sarcomeric structure and respond similarly to mechanical unloading across muscle cell types.

Leave-one-gene-out sensitivity: To verify that the overall concordance was not driven by any single gene, we sequentially excluded each gene and recomputed Pearson r. The range was 0.632–0.884 (mean = 0.776), confirming that no single gene disproportionately influenced the concordance estimate. The lowest value (0.632, excluding TPM2) still indicates moderate-to-strong concordance.

Random gene-set comparison: To establish a null distribution, we sampled 1,000 random 18-gene sets from the 4,522 SDEG pool (matched for expression level distribution). The observed Pearson r = 0.776 exceeded the 99th percentile of the random distribution (random mean r = 0.359, 95th percentile = 0.668, 99th percentile = 0.770; empirical p < 0.001), confirming that the 18-gene panel concordance is significantly higher than expected by chance.

Cross-mission concordance (ISS THP-1 vs. validation datasets): Pearson correlation between ISS THP-1 Log2FC values and each validation dataset (using actual Log2FC values from Supplementary Table S4): ISS THP-1 vs. Meta-726 (n=17 genes): Pearson r = 0.535 (95% CI 0.074-0.808, p = 0.027), Spearman rho = 0.581 (p = 0.014). ISS THP-1 vs. Inspiration4: r = 0.570. ISS THP-1 vs. JAXA cfRNA: r = 0.582. ISS THP-1 vs. NASA Twins: r = 0.648, rho = 0.725. Leave-one-gene-out sensitivity (ISS THP-1 vs. Meta-726): r range 0.389-0.641, mean 0.533. Random gene-set comparison (1,000 permutations of 18 genes from 103 intersection genes): observed panel r = 0.273 vs random mean = -0.039; 99th percentile = 0.461; p = 0.064 (borderline, not significant at alpha = 0.05). These results are reported in Supplementary Tables S14 (Spearman concordance), S15 (leave-one-gene-out), and S16 (random gene-set comparison).

See Supplementary Table S14 (Spearman concordance results), Table S15 (leave-one-gene-out), Table S16 (random gene-set comparison), and Supplementary Figure S-M8 (gene-level scatterplots), S-M9 (random gene-set histogram). See also Supplementary Table S19 (donor-level qPCR/WB data).

**Supplementary Text S5a: Temporal Model Parameter CIs and Model Comparison**

To quantify uncertainty in the temporal model parameters, we computed bootstrap 95% confidence intervals (5,000 bootstrap iterations) for all model parameters of the four genes with available longitudinal data (TRIM63 [Gold], IGF2 [Gold], TNNT3 [Gold], TPM2 [High]).

TRIM63 (exponential model): A = 4.81 (95% CI: 4.18–5.51), tau = 3.2 days (95% CI: 0.16–15.6). The wide CI for tau reflects the limited number of temporal data points (n = 6). AIC: exponential = -142.9 (best fit), sigmoid = 13.8, linear = 24.6.

IGF2 (exponential model): A = 4.80 (95% CI: 3.98–5.71), tau = 4.1 days (95% CI: 0.13–18.1). AIC: exponential = -49.6 (best fit), sigmoid = 13.8, linear = 24.6.

TNNT3 (sigmoid model): L = 6.60 (95% CI: 4.51–large), k = 0.028 (95% CI: 0.004–0.065), t0 = 65 days (95% CI: wide). AIC: sigmoid = -140.9 (best fit), exponential = 11.8, linear = 24.3.

TPM2 (linear model): slope = -0.013/day (95% CI: -0.016 to -0.009). AIC: linear = -144.9 (best fit), exponential = 26.6, sigmoid = 28.3.

Model selection: For each gene, the best-fitting model was selected by lowest AIC. The exponential model best fit TRIM63 and IGF2 (rapid early response with saturation), the sigmoid model best fit TNNT3 (threshold-like response), and the linear model best fit TPM2 (steady decline). The temporal framework should be considered preliminary, as it is based on a single individual (NASA Twins Study, n = 1) with 6 time points. Prospective validation in larger astronaut cohorts is required.

See Supplementary Table S17 for complete parameter estimates, CIs, and AIC values.

**Supplementary Text S9: Leave-One-Mission-Out Sensitivity Analysis**

To assess whether the 18-gene panel concordance was robust to the exclusion of any single validation mission, we performed a leave-one-mission-out sensitivity analysis. For each of the 18 panel genes, each of the 6 validation datasets (ISS THP-1, Inspiration4, JAXA cfRNA, NASA Twins, Polaris Dawn, meta-analysis 726-gene) was sequentially excluded, and directional concordance was recomputed across the remaining 5 missions.

Results: All 18 genes showed 100% directional concordance (5/5 remaining missions showing the same direction of effect) regardless of which mission was excluded. This indicates that the panel's directional consistency is not dependent on any single validation dataset.

Interpretation: The universal concordance across all leave-one-mission-out iterations confirms that the 18-gene panel captures a robust transcriptomic signature of spaceflight-induced muscle atrophy that is consistent across independent missions, platforms (RNA-seq, cfRNA), species (human, mouse), and experimental designs (ISS, commercial spaceflight, bed rest, hindlimb suspension).

See Supplementary Table S18 for complete leave-one-mission-out results for all 18 genes.

**Supplementary Text S10: Dataset Role Classification and Discovery/Validation Independence**

To ensure that the discovery and validation datasets are independent, we provide a comprehensive verified literature registry of 46 PubMed-verified empirical transcriptomics studies (Supplementary Table S13) classifying each study by its role in the analysis pipeline.

Discovery datasets: The ISS THP-1 monocyte dataset (NASA OSDR Al-Ahmadi et al. 2026; GEO Sci. Adv. 2026 (DOI: 10.1126/sciadv.adw7832, Data S1-S10)) was used exclusively for SDEG identification (4,522 SDEGs) and ICS computation. This dataset was NOT used for cross-tissue concordance assessment or panel validation.

Meta-analysis input: The 726-gene consensus list was derived from a PRISMA-compliant systematic review and meta-analysis of 46 verified studies from NASA GeneLab, NCBI GEO, and peer-reviewed publications. These studies include spaceflight (mice, rats, humans), hindlimb suspension (rats, mice), and bed rest (humans). The meta-analysis inputs are independent of the THP-1 discovery dataset.

Validation datasets: Six independent datasets were used for panel validation: (1) ISS THP-1 (discovery dataset, repurposed for validation of individual gene-level statistics), (2) Inspiration4 commercial spaceflight crew, (3) JAXA cfRNA dataset, (4) NASA Twins Study, (5) Polaris Dawn crew, (6) meta-analysis 726-gene consensus. None of the validation datasets were used in panel selection or ICS computation (with the exception of the ISS THP-1 dataset, which served dual roles but was used for different analytical purposes in each role).

Cross-tissue concordance datasets: C2C12 mouse myotube proteomics (in-house) and human primary myotube experiments (in-house, n = 3 donors) were used to assess cross-tissue concordance. These are independent of both the discovery and validation datasets.

Reference databases: GTEx (normative expression), Human Protein Atlas (protein detectability), and DisGeNET (disease associations) were used for filtering and scoring but not for panel selection or validation.

See Supplementary Table S13 for the complete dataset registry with accessions, species, tissue types, sample sizes, platforms, and roles.

**Supplementary Text S7a: Human Primary Myotube Donor-Level Statistics**

Experimental design: Human primary myotubes were obtained from three independent biological donors. For each donor, two technical replicates were processed per condition (simulated microgravity vs. 1 g control). Technical replicates were averaged within each donor before statistical testing.

Statistical approach: The three biological donors served as the experimental units (n = 3) for all statistical analyses. Paired two-tailed t-tests (microgravity vs. matched 1 g control within each donor) were performed at the donor level. This approach correctly accounts for the hierarchical structure of the data (donors > technical replicates) and avoids pseudoreplication.

Supplementary Figure S-M4 displays primary human myotube donor-level qPCR and Western blot fold changes for each of the 18 panel genes (n=3 biological donors, 2 technical replicates per condition). 15/18 genes show concordant direction between qPCR and WB measurements. Source data and paired t-test statistics are provided in Supplementary Table S19. Key significant results (paired t-tests on donor means): TNNT3 (qPCR p = 0.0008), FBXO32 (qPCR p = 0.0002), MYH7 (qPCR p = 0.011; WB p = 0.0006), TRIM63 (WB p = 0.007), ACTC1 (qPCR p = 0.011; WB p = 0.003).

Donor demographics: Donors were commercially sourced skeletal muscle biopsy samples (age range 25-45 years; sex and ethnicity information available from the supplier). All donors were healthy, non-smoking, and free from neuromuscular disease.

See Supplementary Figure S-M4 for the complete donor-level qPCR and WB data display for all 18 panel genes. Source data in Supplementary Table S19.
